# An effective model of endogenous clocks and external stimuli determining circadian rhythms

**DOI:** 10.1038/s41598-021-95391-y

**Published:** 2021-08-09

**Authors:** Tim Breitenbach, Charlotte Helfrich-Förster, Thomas Dandekar

**Affiliations:** 1 Institut für Mathematik, Universität Würzburg, Emil-Fischer-Strasse 30, 97074 Würzburg, Germany; 2 Biozentrum, Universität Würzburg, Am Hubland, 97074 Würzburg, Germany

**Keywords:** Computational biology and bioinformatics, Systems biology

## Abstract

Circadian endogenous clocks of eukaryotic organisms are an established and rapidly developing research field. To investigate and simulate in an effective model the effect of external stimuli on such clocks and their components we developed a software framework for download and simulation. The application is useful to understand the different involved effects in a mathematical simple and effective model. This concerns the effects of Zeitgebers, feedback loops and further modifying components. We start from a known mathematical oscillator model, which is based on experimental molecular findings. This is extended with an effective framework that includes the impact of external stimuli on the circadian oscillations including high dose pharmacological treatment. In particular, the external stimuli framework defines a systematic procedure by input-output-interfaces to couple different oscillators. The framework is validated by providing phase response curves and ranges of entrainment. Furthermore, Aschoffs rule is computationally investigated. It is shown how the external stimuli framework can be used to study biological effects like points of singularity or oscillators integrating different signals at once. The mathematical framework and formalism is generic and allows to study in general the effect of external stimuli on oscillators and other biological processes. For an easy replication of each numerical experiment presented in this work and an easy implementation of the framework the corresponding Mathematica files are fully made available. They can be downloaded at the following link: https://www.biozentrum.uni-wuerzburg.de/bioinfo/computing/circadian/.

## Introduction

Circadian clocks are daily time-keeping mechanisms that help organisms to anticipate the regular 24-h fluctuation on earth. The rhythms generated by circadian clocks are of endogenous nature and persist even in the absence of environmental cues with a species-specific period that usually deviates from 24 h. They are synchronized (=entrained) to the 24-h rhythm on earth by several environmental cues, called Zeitgebers (German for “time-givers”, “synchronizers”), of which light has the largest impact. As nicely summarized in^1^, circadian clocks have the following well defined properties: (1) they entrain to Zeitgeber cycles with a limited range of entrainment, (2) they can follow phase shifts of Zeitgeber cycles, but they need a certain number of transient cycles until they have established their previous phase to the Zeitgeber cycle, (3) they can be phase-shifted by light-pulses in a time-dependent manner that is characterized in a phase-response curve, (4) they free-run under constant conditions with a species-specific period, which is light-dependent. Constant light either lengthens or shortens the free-running period and constant light of high intensity dampens the clock and finally leads to arrhythmicity. Our aim is to start from a known mathematical oscillator model based on experimental findings and extend it to an effective modeling framework for the circadian clock that includes the impact of external stimuli.

Although the general properties of circadian clocks are the same in all organisms, species-specific differences exist. When comparing for example the mouse circadian clock with the fruit fly circadian clock, which are standard models for mammalian and insect clocks, the following differences become evident: (1) The mouse (*Mus musculus*) clock has a narrower range of entrainment and it needs longer to follow phase-shifts of the Zeitgeber cycle than the fruit fly (*Drosophila melanogaster*) clock. This suggests that it is less plastic and less light-sensitive. (2) In line with this suggestion, the mouse phase-response curve to light has a lower amplitude than that of the fruit fly and the mouse free-running period is less responsive to the light intensity. Our framework presents an effective model covering all these variants of a circadian clock.

Some of these differences can be explained on the molecular level. In both model systems, circadian rhythms are generated by molecular negative feedback loops in which the gene products of certain clock genes inhibit their own transcriptional activators after a time delay (reviewed in^2, 3^). The orthologous transcriptional activators are called CLOCK and BMAL1 in *Mus* and CLOCK and CYCLE in *Drosophila*. They form heterodimers that activate transcription of the genes *cryptochrome 1* and *2* (*cry1/2*) and* period 1* and *2 *(*per1/2*) in *Mus* and *period* (*per*) and *timeless* (*tim*) in *Drosophila*. PER1/2-mCRY1/2 protein complexes in *Mus* and PER– TIM protein complexes in *Drosophila* are modified (e.g. phosphorylated) and after a time delay inactivate the CLOCK-BMAL1 and CLK-CYC activators, respectively, to repress transcription. PER1/2-CRY1/2 and PER-TIM complexes are subsequently degraded, which permits the activators to initiate the next cycle of transcription. We solve this in one framework by adding corresponding terms to existing clock models denoted in Table 1.

A main difference exists in the effects of light on this negative feedback loop: whereas light induces the transcription of* per1/2* in the mouse, light leads to the degradation of TIM in the fruit fly (reviewed in^2^). The higher light sensitivity of the fruit fly clock can be explained by the presence of a light-sensitive form of Cryptochrome (*Drosophila* CRY) that is not part of the negative feedback loop but instead interacts with TIM in the presence of light^4, 5^. *Drosophila* CRY is expressed in the clock neurons and leads to an immediate degradation of TIM and a reset of the clock in the presence of light^6^. This is true for all tissues in which the clock is ticking, not only the master clock in the brain^7^. Thus, the master clock in the brain and the body clocks run with the same phase and both are quickly entrainable by light. In contrast, mouse CRYs are light-insensitive and the information about light has to reach the master clock in the brain, specifically the suprachiasmatic nucleus (SCN), via the eyes and the retinohypothalamic tract (reviewed by^8^). The mouse master clock in the SCN then synchronizes the clocks in the body. Thus, there are several steps between light reception and entrainment of the master clock as well as between entrainment of the master clock and the body clocks, also called peripheral oscillators. The peripheral oscillators are not only entrainable by the master clock, but in addition by other Zeitgebers like feeding^9^. As a consequence the peripheral oscillators are phase-delayed by different time shifts with respect to the master clock^10, 11^. The main focus of the presented work is to show how to systematically generate sub models and combine them to a total model demonstrated by coupling these different oscillators.

More specific, the aim of the present work is hence to investigate a purposeful framework to include external stimuli into a model and thus generate a systematic way to couple models with an input-output-interface. We show the usability with an effective model of the circadian clock since the circadian clock is affected by many external stimuli. Moreover, as mentioned, the inner clock of an organism consists of many coupled oscillators, see e.g.^11, 12^, which is a clear use case for an input-output-interface to couple all the sub clocks since well validated models can easily be connected to build complex models and analyze them. A module wise modeling procedure, where for the modeling of each sub system powerful data driven model approaches are available like vector field optimization^12^ or Potterswheel^13^, allows a quick investigation which model might be improved to enhance the performance of the compound model. This strategy keeps complex models understandable and handy. For the purpose of demonstration, we use a model with minimal components necessary to reproduce the basic observed model behavior (see experiments below, e.g. Table 1), in particular a 24 h oscillation, to keep the discussion clear and focused to the discussion of the input-output-framework with external stimuli. Our model starts from the formalism and original model of Goldbeter^14^, since the model is derived from molecular mechanisms which allows to show some biologically motivated modeling issues, see e.g. section “Specific organism models". The Goldbeter model is extended with an input-output-framework with external stimuli. We show and focus on the properties and interactions in this system which are critical to reproduce, study and analyze important system behavior of circadian clocks as summarized in Table 1 where the investigations with an uncoupled model are basically used to show either modeling issues or to validate that the proposed external stimuli framework is able to simulate known capabilities that have already been studied, see the “Discussion” for details. The presented framework is not limited to the Goldbeter model. Any model consisting of differential equations can be taken, see Table 2 for some examples of suitable chronobiological models, where only the model equations need to be exchanged in our provided Mathematica files with the one referenced in the column “Model equations”.

In our work, we extend this model with additional terms modeling specific effects from different origin. Table 1 gives an overview over the simulated experiments and where the data can be found in the present work. We remark that we have to be careful with external stimuli that change reaction parameters of the model^14^ noteworthy, like temperature or certain chemical agents, since the model of Goldbeter is based on enzyme kinetics where model parameters, like reaction coefficients, depend on temperature for example. These parameters are assumed to be constants for the scope of the work which is to discuss the input-output-interface of models. However it paves the way for extension by letting the “constants” depend on temperature or on other chemicals like ions in case a more detailed model is needed. The effect of temperature and chemicals can also be modeled by including corresponding enzyme kinetic terms into the model instead of changing model parameters where the effect of the temperature or chemicals act as the external stimuli.

In the present work we focus on establishing a general framework for the effective inclusion of external stimuli. That means how to include only necessary information of the effect of external stimuli on the agents of a biological system to be able to explain the effect of external stimuli on the behavior sufficiently well. This framework has already been used to extend SQUAD models for regulatory networks^15, 16^. Now, we demonstrate the wide applicability of the proposed framework, in particular to models for enzyme kinetics, exemplary based on the model of Goldbeter^14^, which is without temperature dependency of the parameters, by reproducing many experimental outcomes with a single external stimuli framework, see Table 1. This works because a lot of experiments are done with light, where temperature is constant during the experiments or chemicals used do not change the reaction parameters of the enzymes for the molecular clock.

**Table 1:** The table summarizes experiments with corresponding literature and where the covering in the presented work can be found. In the column “experiment” we give a keyword describing the experiment, in “exp. evidence” we refer to literature publishing data to the corresponding experiment and in “in presented work” we show where we can find these experiments in the presented work.

Experiment	Exp. evidence	In present work
Synchronization to external Zeitgeber	^17^	Figure 2a,c,e, Supplementary Figure 4
Restart of the endogenous clock	^18^	Supplementary Figure 3
Entrainment to different external periods	^19^	Paragraph following (21)
Stimulus of constant intensity (Aschoff’s rule)	^20, 21^	Figure 4e
Phase-response curves	^22–24^	Supplementary Figure 7e
Synchronization of peripheral clocks by the SCN	^9, 11, 12, 25^	Figures 5a,c,e, 6
Points of singularity	^26^	Discussion (page 20)

The focus of this work is not on calculating all specific effects in Table 1 in detail but introducing a framework and a systematic procedure that is able to include all these effects and simulate them. An advantage of our framework of including external stimuli is that it is independent of the model chosen. There is a considerable body of literature making the biological model of the circadian clock more complete, for instance studying the specific components present in the fly or mammalian circadian clock (see Table 2). The simulation is then easily extended including these proper formulas in our Mathematica framework. For example, the ideas presented in this work how to include external stimuli into circadian clock models can easily be extended to more complex models of the fly model shown in^27^. With analogous terms as presented in this work where we let light act on PER we can model the effect of light on the expression of TIM. The terms which model the external stimuli perform their effect by increasing or decreasing the level of expression of the corresponding gene. The same works for models that are for mammalians. There are models ranging from basic^28–31^ to complex^32–34^ where in particular PER and CRY interactions are modeled. Also in this case the corresponding equations that model the expression of, e.g., PER and CRY are supplemented by the same terms that are presented in this work in order to influence their expression by the action of the modeled stimuli. What makes all these models equivalent for the scope of this work is that they all have oscillations with a 24 h period where we for the purpose of clarification choose one with only a few equations. More specific, mathematical models can be used to test the hypothesis if a model can explain the measurement, e.g. a molecular measurement of genes and can extend such model by for example extend feedback loops. However, for some research questions, also effective models are necessary, for example where just the effect of a feedback loop is important and not all the details of involved agents. Another focus of the paper is to provide a framework for including external stimuli and input-output-interfaces for coupling oscillators. Consequently, the framework also works for (non-)linear (micro-)oscillator coupling models where examples for (micro-)oscillators can be found in Table 2 but the framework is not limited to them.

Once the external stimuli are included, we can analyze the model with respect to an optimal interaction with the external stimuli as shown in^15, 16^ in order to influence the circadian clock and thus the cell behavior. This is in particularly attractive if we think of external stimuli as drugs that interact with the circadian clock in order to calculate optimal therapies for which our proposed method how to include external stimuli can also be used. The concept of chronotherapy, meaning paying more attention to the circadian rhythm for therapies, in particular a time depending administration of drugs, is already mentioned in^10^. By unified modeling strategies the information encoded in a circadian model can efficiently be used in optimization frameworks (e.g.^15, 16^) to accelerate the development of drugs by calculating optimal points of times for drug administration and such safe time by validating a promising candidate from a computation with an experiment shortening the phase of (re)search.

**Table 2:** In this table there is a selection of model equations that can be chosen for the combination with our external stimuli framework instead of the Goldbeter model used in this work by substituting the model equations in the provided Mathematica files with the equations referenced in the column “Model equations”. In the column “Literature” there is the reference of the publication with the year of publication and in the column “Description” we give a keyword describing the presented model.

Literature	Model equations	Description
^35^ (1999)	(1)–(2)	Basic oscillations
^30^ (1994)	(2), (3), (4)	Intercommunication
^36^ (2011)	Table 2	Mathematical oscillator models
^27^ (1998)	(1a)–(1j)	PER/TIM complex
^33^ (2003)	Supporting text	Mammalian circadian clock
^34^ (2003)	Appendix 1	Detailed mammalian model
^12^ (2018)	Supplementary information 1	Delay-differential-equations (DDE)
^37^ (2016)	S1 Appendix	DDE, Repressilator
^38^ (2016)	Supplemental information	Metabolism mammalian clock
^39^ (2019)	Equation (3)	Plant (*Arabidopsis*) circadian clock (DDE)

However, to derive such a more complex and detailed model of the circadian clock is not the aim of the present work. The aim is to establish a systematic input-output-interface demonstrated on the topic of circadian clock. This formalism has a systematic bilinear structure where the function of the external stimuli is multiplied by another quantity. According to the law of mass action our presented formalism is a building block for more complex situations and thus we present a rational approach to model biological systems with as many equations as necessary to capture effects like cooperativity or until the model cannot be rejected based on experimental data. By the possibility of considering external stimuli as input functions for models, we show how to couple different models by replacing the external stimuli by the corresponding output functions of another model, as presented in section “Modeling central and peripheral oscillators” and section “Food entrained peripheral clocks”. In this way, we can test and refine each single (sub) model for small biological systems and consequently have building blocks for constructing big and thus complex biological models by well validated small models. This usually makes the procedure of modeling more efficient since the parts of the models that possibly need to be refined can be identified fast and adjusted for the corresponding situation. For this purpose, the external stimuli of a sub model and its output need to be measured and validated if the simulated output fits sufficiently well to the measured output, which is the experimental data.

In order to describe the circadian clock modeling framework, we start from the basic model of Goldbeter which we introduce in the following. Subsequently, we give an introducing example entraining the model to light dark cycles. After discussing some modeling issues to adapt the model to different species, we show results oft the model under constant light and validate these results with experimental results from literature. Then we show how to combine these basic models to generate a total model of synchronizing different clocks in an organism. In the Supplementary material, we present additional basic experiments of a single oscillator/model that we found in the literature to further validate our external stimuli framework by showing that we obtain the same results. Furthermore, the examples of the Supplementary material demonstrate how to model different effects with our external stimuli framework to become more familiar with the framework. In the “Discussion”, we relate our work to existing publications and give an outlook for future work by discussing an explanation of singularity points (one pulse of light can stop the oscillation and another can start the oscillation again, see^26, 40^) with our model framework.

For an easy replication of each numerical experiment presented in this work and an easy implementation of the framework the corresponding Mathematica files are fully made available. They can be downloaded at the following link: https://www.biozentrum.uni-wuerzburg.de/bioinfo/computing/circadian/.

## Results

We recall the basic model of Goldbeter before we demonstrate how to couple different oscillators.

## The model of Goldbeter

**Figure 1:**
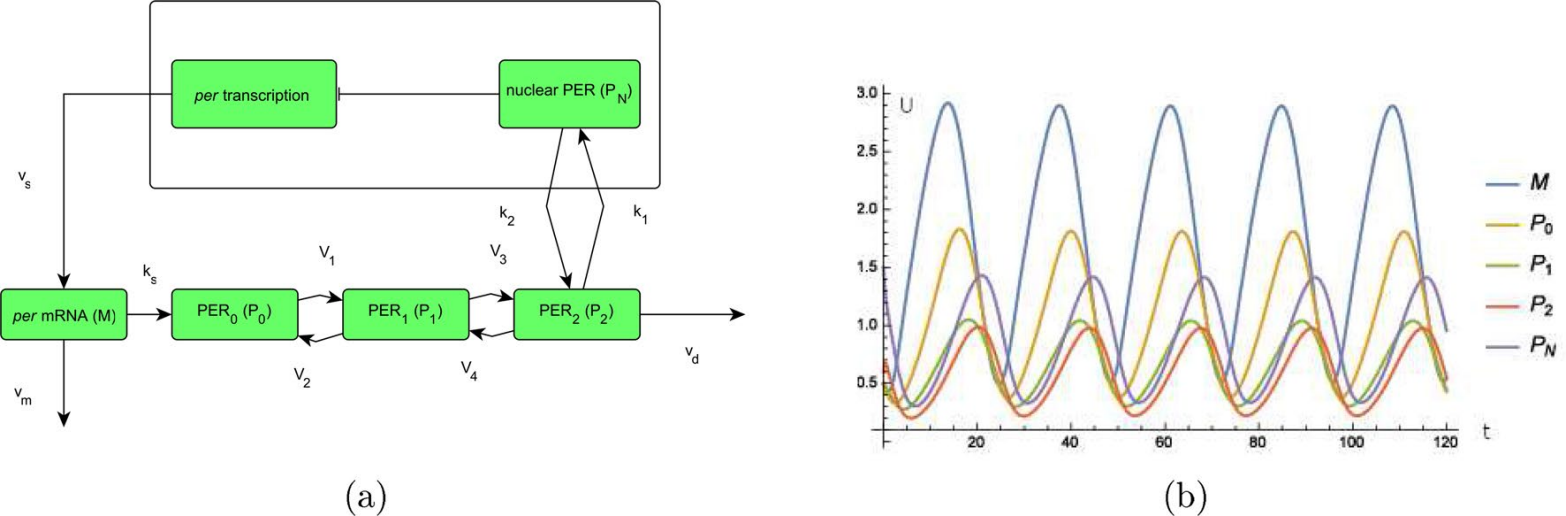
(**a**) Model for circadian PER oscillations, see^14^. Scheme of the model for circadian oscillations of PER and *per* mRNA. The system of the equations is given by (1) to (5). The *per*mRNA (*M*) is transcribed in the nucleus and is transported to the cytosol, where it accumulates at a maximum rate $v_{s}$; thereby it is degraded by an enzyme of maximum rate $v_{m}$ and Michaelis constant $K_{m}$. The rate of translation of the PER protein, that is proportional to *M*, is characterized by a first-order rate constant $k_{s}$. The parameters $V_{i}$ and $K_{i}$, $i=1,2,3,4$, denote the maximum rate and Michaelis constant of the kinase(s) and phosphatase(s) involved in the reversible phosphorylation of $P_{0}$ into $P_{1}$ and $P_{1}$ into $P_{2}$ of the PER protein, respectively. The fully phosphorylated form ($P_{2}$) is degraded by an enzyme of maximum rate $v_{d}$ and Michaelis constant $K_{d}$, and transferred into the nucleus at a rate characterized by the first-order rate constant $k_{1}$. Transport of the nuclear, biphosphorylated form of PER ($P_{N}$) into the cytosol is characterized by the first-order rate constant $k_{2}$. The negative feedback exerted by nuclear PER on *per* transcription is described by an equation of the Hill type, in which *n* denotes the degree of cooperativity and $K_{I}$ the threshold constant for inhibition. (**b**) A plot of the model (1) to (5) with the parameters $v_{s}=0.76\frac{\mu \hbox {M}}{h}$, $v_{m}=0.65\frac{\mu \hbox {M}}{h}$, $K_{m}=0.5\mu \hbox {M}$, $k_{s}=0.38\frac{1}{h}$, $v_{d}=0.95\frac{\mu \hbox {M}}{h}$, $k_{1}=1.9\frac{1}{h}$, $k_{2}=1.3\frac{1}{h}$, $K_{I}=1\mu \hbox {M}$, $K_{d}=0.2\mu \hbox {M}$, $n=4$, $K_{1}=K_{2}=K_{3}=K_{4}=2\mu \hbox {M}$, $V_{1}=3.2\frac{\mu \hbox {M}}{h}$, $V_{2}=1.58\frac{\mu \hbox {M}}{h}$, $V_{3}=5\frac{\mu \hbox {M}}{h}$, $V_{4}=2.5\frac{\mu \hbox {M}}{h}$ with the initial values $M\left( 0\right) =0.5$, $P_{0}\left( 0\right) =0.5$, $P_{1}\left( 0\right) =0.5$, $P_{2}\left( 0\right) =0.6$, $P_{N}\left( 0\right) =1.5$. Units and dimensions: abscissa *t*: time in hours, ordinate *U*: amount of substance (*M* mRNA, $P_{i}$, $\left\{ 0,1,2,N\right\} ,$ protein).

In the work of^14^, a model based on enzyme kinetics and ordinary differential equations is described that provides limit cycles and thus can be used to model endogenous oscillations of proteins in a cell. Specifically, that model describes the oscillations of the *Drosophila* period protein (PER) and its multiple phosphorylation. A schematic of the network and a detailed description is given in Fig. 1a and the corresponding system of ordinary differential equations is given by

1 $$\begin{aligned} \frac{d}{dt}M&=v_{s}\frac{K_{1}^{n}}{K_{I}^{n}+P_{N}^{n}}-v_{m}\frac{M}{K_{m}+M} \end{aligned}$$

2 $$\begin{aligned} \frac{d}{dt}P_{0}&=k_{s}M-V_{1}\frac{P_{0}}{K_{1}+P_{0}}+V_{2}\frac{P_{1}}{K_{2}+P_{1}} \end{aligned}$$

3 $$\begin{aligned} \frac{d}{dt}P_{1}&=V_{1}\frac{P_{0}}{K_{1}+P_{0}}-V_{2}\frac{P_{1}}{K_{2}+P_{1}}-V_{3}\frac{P_{1}}{K_{3}+P_{1}}+V_{4}\frac{P_{2}}{K_{4}+P_{2}} \end{aligned}$$

4 $$\begin{aligned} \frac{d}{dt}P_{2}&=V_{3}\frac{P_{1}}{K_{3}+P_{1}}-V_{4}\frac{P_{2}}{K_{4}+P_{2}}-k_{1}P_{2}+k_{2}P_{N}-v_{d}\frac{P_{2}}{K_{d}+P_{2}} \end{aligned}$$

5 $$\begin{aligned} \frac{d}{dt}P_{N}&=k_{1}P_{2}-k_{2}P_{N} \end{aligned}$$

where *M* is the amount of substance of *per* mRNA in the cytosol, $P_{0}$ is the amount of substance of unphosphorylated, $P_{1}$ the amount of substance of monophosphorylated, $P_{2}$ the amount of substance of biphosphorylated PER protein, $P_{N}$ is the amount of substance of the biphosphorylated PER protein in the nucleus.

**Figure 2:**
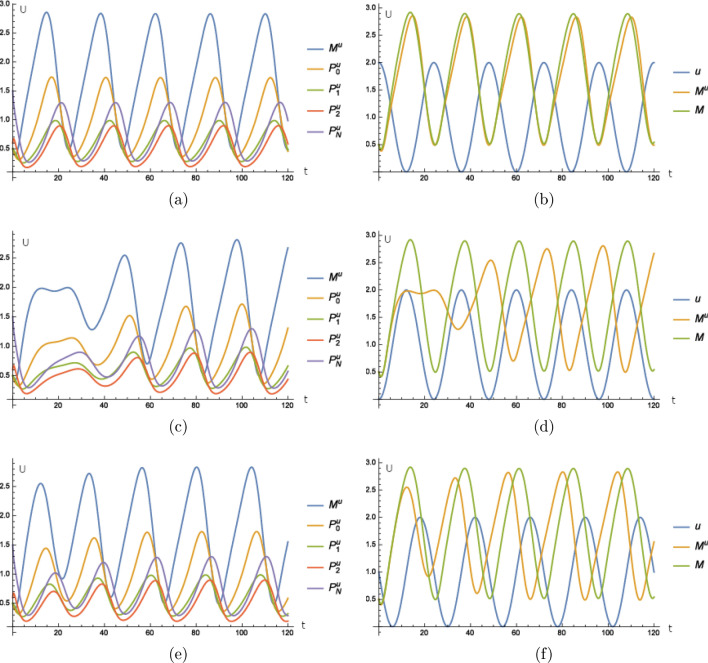
The model of Goldbeter including our extension does not differ from the original model of Goldbeter if the molecular clock is in the phase of the external Zeitgeber. (**a**) Time curves where the superscript *u* indicates that these time curves stem from the extended model consisting of (6) and (2) to (5) with (7) for $\varphi =0$ and $\alpha =0.05$. (**b**) Time curve of the external stimulus *u* defined in (7), of *M* calculated from the model (1) to (5) and of $M^{u}$ calculated from (6) and (2) to (5) with (7) for $\varphi =0$ and $\alpha =0.05$. For the figure (**c**) and (**d**) the external stimulus induces a shift of the molecular clock’s phase of 12 h where the experiment is as in (**a**) and (**b**) but with $\varphi =\pi$. The external stimulus induces a shift of the molecular clock’s phase of about 6 h in (**e**) and (**f**) where the experiment is as in (**a**) and (**b**) with $\varphi =\frac{\pi }{2}$. Units and and dimensions: abscissa *t*: time in hours, ordinate *U*: amount of substance (*M*, $M^{u}$ mRNA, $P_{i}^{u}$, $\left\{ 0,1,2,N\right\} ,$ protein, *u* intensity of illumination).

Also in the work of^14^, the model used in the present work is validated with experimental results. For our calculation with the model (1) to (5) we use the following values for the parameters which can be found in^14^, Figure 2. We have $v_{s}=0.76\frac{\mu \hbox {M}}{h}$, $v_{m}=0.65\frac{\mu \hbox {M}}{h}$, $K_{m}=0.5\mu \hbox {M}$, $k_{s}=0.38\frac{1}{h}$, $v_{d}=0.95\frac{\mu \hbox {M}}{h}$, $k_{1}=1.9\frac{1}{h}$, $k_{2}=1.3\frac{1}{h}$, $K_{I}=1\mu \hbox {M}$, $K_{d}=0.2\mu \hbox {M}$, $n=4$, $K_{1}=K_{2}=K_{3}=K_{4}=2\mu \hbox {M}$, $V_{1}=3.2\frac{\mu \hbox {M}}{h}$, $V_{2}=1.58\frac{\mu \hbox {M}}{h}$, $V_{3}=5\frac{\mu \hbox {M}}{h}$, $V_{4}=2.5\frac{\mu \hbox {M}}{h}$ with the initial values $M\left( 0\right) =0.5\ \mu \hbox {M}$, $P_{0}\left( 0\right) =0.5\ \mu \hbox {M}$, $P_{1}\left( 0\right) =0.5\ \mu \hbox {M}$, $P_{2}\left( 0\right) =0.6\ \mu \hbox {M}$, $P_{N}\left( 0\right) =1.5\ \mu \hbox {M}$. We always calculate the model from time $t=0$ to a final time. The calculations in this work are performed with Wolfram Mathematica. In Fig. 1b, we have a plot of the model where we see an almost 24 h rhythm analogous to^14, Figure 2]^. Throughout the work the time is plotted on the abscissa and the corresponding amount of response is plotted on the ordinate if not otherwise stated. The physical units and dimensions of the response measured are given in the figure legend. As pioneered by the work of Goldbeter, the parameters above are chosen such that an approximately 24h period is obtained in the model (1) to (5) and the units are provided tentatively. Consequently, the discussions in this work can also be done for concentration referred to a certain volume, e.g. cell volume, as a unit for mRNA and proteins instead of amount of substance. However, the framework is not limited to molecular oscillators, like also oscillators for electrical waves could be considered for instance.

In the following, we introduce a systematic procedure that results in effective models that include the interaction of external stimuli with a studied biological system. For the demonstration of the practicability of this procedure we give several examples including reproduction of known results from different models and new model possibilities that are provided by our approach of considering external stimuli. For this purpose, we include bilinear control terms in order to alter the corresponding state quantities of the core model.

## Entrainment to light–dark cycles

We extend the model of^14^ by a mechanism that allows to include external stimuli. The idea is that the external stimulus affects the corresponding agent to whose model equation it is applied such that it either increases or decreases the rate which the value of the considered agent changes according to its model. We know that external stimuli influence the circadian clock and can synchronize the rhythm to the external time phase. Such an external stimulus is for example light, see^17^ or any other substances that influence the concentration of an agent of the model (1) to (5). An important application aspect is that tolerance to harsh pharmacological treatment such as bone marrow transplantation, radiation or mitigation of graft versus host disease depends on the time of the day. Knowing the influence of external stimuli on the circadian clocks is hence important for any pharmacological treatment, in particular at high dose treatment regimes, but is usually not attempted as a general framework for modeling such influences is lacking. This is, however, provided here.

In our first experiment, we synchronize the phase of the endogenous clock to the phase of an external Zeitgeber. We start our discussion by introducing now external influences changing the circadian oscillator. A simple influence assumes that the external stimulus leads to a further decay of per mRNA (M) (e.g. achieved by expressing a miRNA in the system or a treatment via any suitable drug). Since this is an effective model, we can just as well model the effect on the protein concentration with a similar effect. We model the further decay of *per* mRNA by

6 $$\begin{aligned} \frac{d}{dt}M&=v_{s}\frac{K_{1}^{n}}{K_{1}^{n}+P_{N}^{n}}-v_{m}\frac{M}{K_{m}+M}-\alpha uM \end{aligned}$$

instead of (1). The term $-uM$ models the effect that if the external stimulus is strong and *M* is big, then the decay of *M* is strong, that means a lot of *per* mRNA is degraded and if the external stimulus is weak and *M* is low, then there is almost no additional decay of *M*. The term *uM* is called a bilinear term since the effect of the stimulus and of the agent, which the stimulus effects, are multiplied in contrast to linear terms where just the term *u* is considered. The constant $\alpha$ is a coupling constant that weights the influence of the term $-uM$ on the time variation of *M*. For our experiment, we use $\alpha =0.05$.

Our first experiment is as follows. We assume light as our periodic external stimulus and we assume that the effect of this stimulus can be effectively modeled by an increased degradation of *per* mRNA. The light intensity oscillates with a period of 24 h according to

7 $$\begin{aligned} u\left( t\right) {{:}{=}}\cos \left( 2\pi \frac{t}{24}+\varphi \right) +1 \end{aligned}$$

where $\varphi$ is the time shift of external and internal time of the cell. We remark that there are more sophisticated ways to model the intensity of light, for example with a constant 0 over night and a peak about noon. However, this does not change the qualitative arguments in our analysis while making the study more complex. We start with $\varphi =0$. Then, the external stimulus has the same phase as the endogenous time of the cell, see Fig. 2a,b and the curves almost look like the unperturbed ones from Fig. 1b. That means that our framework is in accordance with the model proposed in^14^. Furthermore, we see that the period stays the same and is not changed by the mechanism that includes the external stimulus. Regarding the different forms of PER, we only show a detailed plot of the mRNA (Fig. 2b) since the plots of all forms look the same. This is done throughout the following work. Later in the paper we discuss the consequences of the external period differing from the period of the endogenous clock.

If the external time is shifted compared to the endogenous time of the cell by for example $\varphi =\pi$, then we see in Fig. 2c,d that the endogenous time is shifted by approximately 12 h after a transient of about 2 days. The external stimulus synchronizes the endogenous clock with the external time so that the phase of the cell’s endogenous time is again in sync with the phase of the external time. We remark that the period of about 24 h is unperturbed after the transient.

For illustration, we have the figures analogous to the results above for $\varphi =\frac{\pi }{2}$ and have a shift of about 6 h after a transient, see Fig. 2c,f.

## Specific organism models

**Figure 3:**
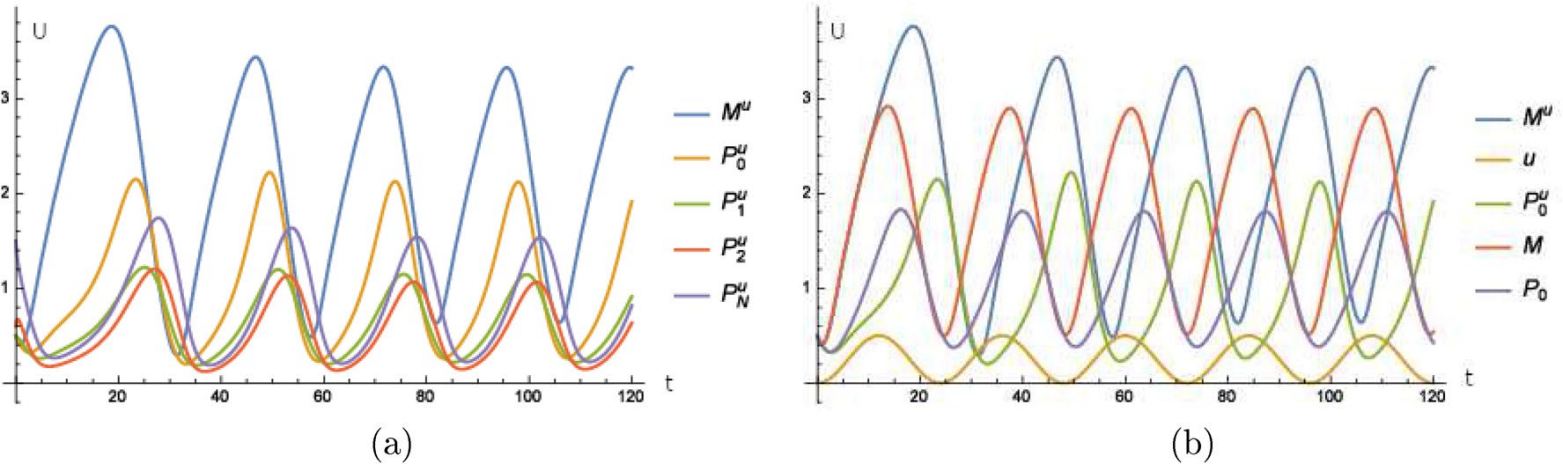
A further digestion of PER protein shifts the phase of the molecular clock. (**a**) Time curves where the superscript *u* indicates that these time curves stem from the extended model consisting of (1) to (5) where (2) is replaced by (8) for (16) and $\alpha =1$. (**b**) Time curve of the external stimulus *u* defined in (16), of *M* and $P_{0}$ calculated from the model (1) to (5) and of $M^{u}$ and $P_{0}^{u}$ calculated from (1) to (5) where (2) is replaced by (8) for (16) and $\alpha =1$. Units and dimensions: abscissa *t*: time in hours, ordinate *U*: amount of substance (*M*, $M^{u}$ mRNA, $P_{i}^{u}$, $\left\{ 0,1,2,N\right\}$, protein, *u* intensity of illumination).

In the fly we have, mediated by a signal pathway, a degradation of the PER protein caused by the external stimulus effectively modeled with

8 $$\begin{aligned} \frac{d}{dt}P_{0}=k_{s}M-V_{1}\frac{P_{0}}{K_{1}+P_{0}}+V_{2}\frac{P_{1}}{K_{2}+P_{1}}-\alpha uP_{0} \end{aligned}$$

where $\alpha >0$. We take our minimal model (1) to (5) where (2) is exchanged by (8) for flies. We show that, analogously to Supplementary Figure 4, the unperturbed rhythm is shifted by about 12 h if one applies (16) with $\varphi =\pi$, see Fig. 3.

In mammals we have instead that light induces* per *transcription. Therefore, we introduce a new model where we replace (1) by

9 $$\begin{aligned} \frac{d}{dt}M=v_{s}\frac{K_{1}^{n}}{K_{1}^{n}+P_{N}^{n}}-v_{m}\frac{M}{K_{m}+M}+\gamma u\exp \left( -\varrho M\right) \end{aligned}$$

while we still use (2) to (5) with $\gamma ,\varrho >0$. The term $\exp \left( -\varrho M\right)$ models the fact that if there is a lot of *per* mRNA then a light stimulus is supposed to be not that effective as it is when there is almost no *per* mRNA. In this work, we use $\varrho =1$.

We remark that we assume that the production of mRNA (*M*) is limited by the capacity of the transcription process. That means once this limit is reached more strength of external stimuli has no effect. We assume that $\exp \left( -\varrho M\right)$ models the fact that if there is much *M*, then there will be a lot of active transcription machinery. If we would like to avoid the exponential term and replace it with a bilinear mechanism with the same qualitative effect, we have to add a further equation modeling the transcription capacities. For example we have the species *T* which is the unstimulated transcription machinery and $T^{*}$ which is stimulated one by the external stimulus. We have $\frac{d}{dt}T=-\varrho uT+\xi T^{*}$ and $\frac{d}{dt}T^{*}=\varrho uT-\xi T^{*}$ with $T\left( 0\right) =T_{0}>0$, $\xi >0$ and $T^{*}\left( 0\right) =0$ for example. The external stimulus *u* transforms the unstimulated species into the stimulated one. The term $\xi T^{*}$ models the decay of $T^{*}$ or a self inhibition. By the fixed initial value $T_{0}$, the capacity of transcription machinery that can be stimulated is limited and we can replace $u\exp \left( -\varrho M\right)$ by $T^{*}$ in (9). Of course we can further extend this model by including a negative feedback loop into the equations for *T* and $T^{*}$ which models the fact that mRNA limits its own transcription. This could be done be adding $MT^{*}$ into the equation for *T* and subtract it in the equation for $T^{*}$ where each $MT^{*}$ is multiplied with the same constant. Depending on the quality of the data, one may also model the reaction mechanisms more detailed if there are several molecules binding to certain proteins of the transcription machinery or if there are cascades of reactions where different molecules bind to the same protein one after another. The smaller the error bars of the data points are, the more sophisticated models, i.e. with more parameters, can be tested and distinguished with respect to if they have to be rejected based on the data or not. To implement more sophisticated models we can use modified equations describing the dynamics according to the law of mass action for each species defined by the molecules that have already bound to the corresponding protein. Since the focus of this work is on the presentation of working systematically with external stimuli where all the previous discussed formulas fit into, we would like to proceed with the most easy model presented in (9) for reasons of clarity.

Another modeling case could be that the external stimulus *u* itself is included into a non-linear function, e.g. $u^{2}$. A motivation for such a term can be cooperativity effects of two molecules activated by light. This can be transferred to the following system of equations which all have again terms that are linear in the external stimulus term and consequently again bilinear as a product of agent and stimulus. We have the following sub system

$$\begin{aligned} \begin{aligned}\frac{d}{dt}M_{1}&=uM_{1}-\gamma ^{h}M_{1}\\ \frac{d}{dt}M_{2}&=uM_{2}-\gamma ^{h}M_{2} \end{aligned} \end{aligned}$$

where *u* is the original external stimulus and the initial values of $M_{1},M_{2}$ and the decay rate $\gamma ^{h}$ are greater than zero. According to the approach of the law of mass action the former term $u^{2}$ would now be expressed by $M_{1}M_{2}$ in which the quadratic effect of $u^{2}$ in the original equation is covered.

## Constant light

How do we model a constant stimulus such as light? We choose a constant external stimulus, i.e.

10 $$\begin{aligned} u_{c}\left( t\right) =c, \end{aligned}$$

that causes a degradation of $P_{0}$ (fly model) or a further transcription of *M* (mouse model) where $c\ge 0$ is the intensity of the external stimulus. We determine the periods by the peak of the oscillations of the *per* mRNA determined with the FindArgMax function of Mathematica in the range $t\in \left[ 80,120\right]$ hours.

**Figure 4:**
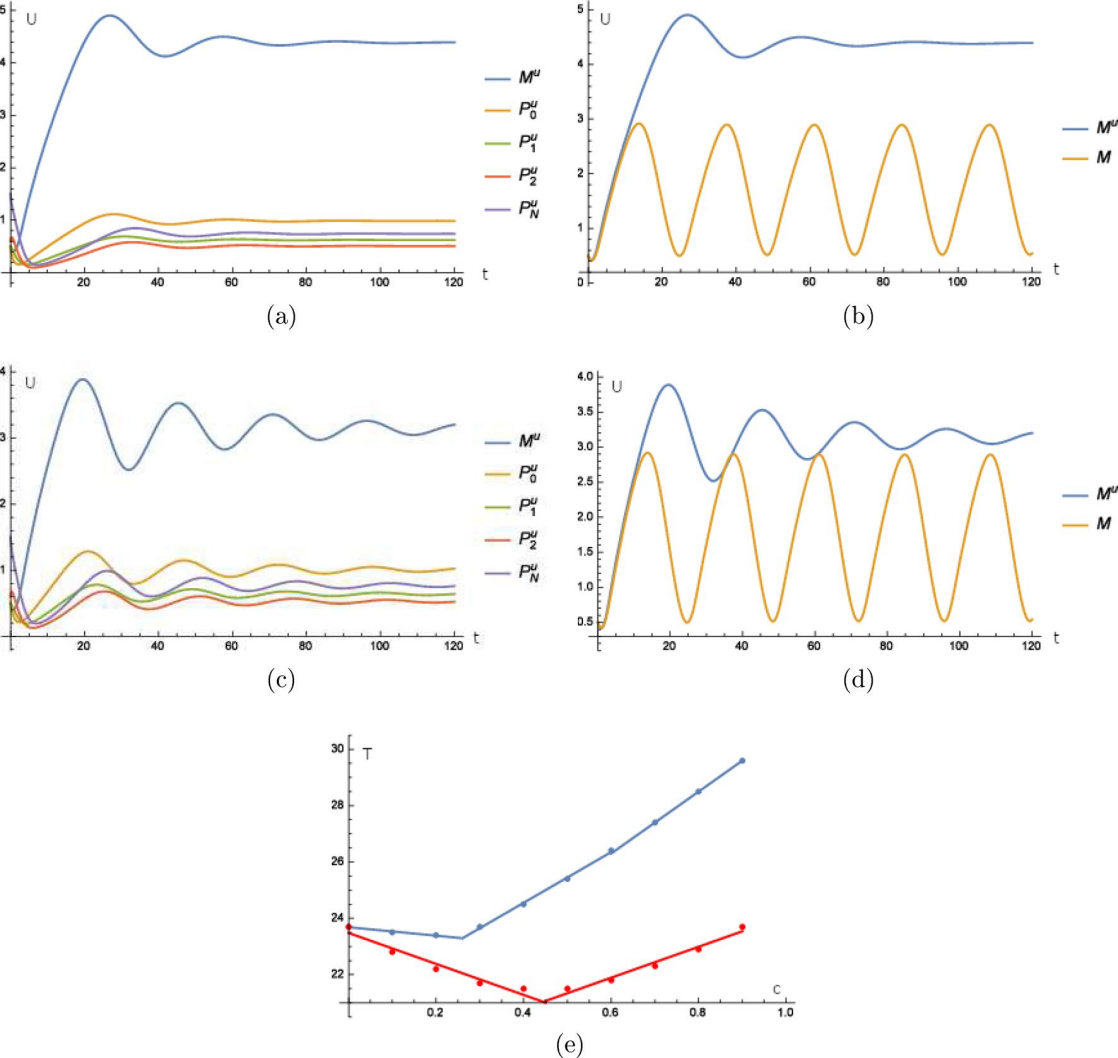
Aschoff’s rule: a strong permanent external stimulus causes arrhythmic behavior as the oscillations are dampened off. (**a**) Time curves where the superscript *u* indicates that these time curves stem from the extended model consisting of (1) to (5) where (2) is replaced by (8) for (10) with $\alpha =1$ and $c=1$. (**b**) Time curve of *M* calculated from the model (1) to (5) and of $M^{u}$ calculated from (1) to (5) where (2) is replaced by (8) for (10) with $\alpha =1$ and $c=1$. In (**c**) and (**d**) a weak permanent external stimulus causes dampening of the amplitude of the oscillations, however they are still measurable. The experiment is as in (**a**) and (**b**) with $c=0.5$. Units and dimensions for (**a**)–(**d**): abscissa *t*: time in hours, ordinate *U*: amount of substance (*M*, $M^{u}$ mRNA, $P_{i}^{u}$, $\left\{ 0,1,2,N\right\} ,$ protein, *u* intensity of illumination). In (**e**) we have on the abscissa the illuminance *c* and on the ordinate we have the period length *T* in hours. The periods of the endogenous clocks of the fly and the mammalian model under a constant external stimulus. The blue graph is from the fly model and the red one is from the mammalian model. The data points for the fly model are $\left( 0,23.7\right)$, $\left( 0.1,23.5\right)$, $\left( 0.2,23.4\right)$, $\left( 0.3,23.7\right)$, $\left( 0.4,24.5\right)$, $\left( 0.5,25.4\right)$, $\left( 0.6,26.4\right)$, $\left( 0.7,27.4\right)$, $\left( 0.8,28.5\right)$, $\left( 0.9,29.6\right)$. The data points for the mammalian model are $\left( 0,23.7\right)$, $\left( 0.1,22.8\right)$, $\left( 0.2,22.2\right)$, $\left( 0.3,21.7\right)$, $\left( 0.4,21.5\right)$, $\left( 0.5,21.5\right)$, $\left( 0.6,21.8\right)$, $\left( 0.7,22.3\right)$, $\left( 0.8,22.9\right)$, $\left( 0.9,23.7\right)$.

We start with the fly model with $\alpha =1$ and obtain first a shortening of the period and from a turning point an elongation of the period the stronger the stimulus becomes until we have no oscillation any more which we call arrhythmic behavior. See Fig. 4a,b for a strong intensity where $c=1$ causes an arrhythmic behavior, which means that the oscillations fade for a strong intensity of the external stimulus until there is no rhythm, and see Fig. 4c,d for a weak intensity of the external stimulus where $c=0.5$ and the amplitude of the oscillations is smaller compared with the unperturbed oscillations.

Now, we investigate the mammalian model for $\gamma =2$. This causes that the model also shows arrhythmic behavior for $c=1$. We have that the period becomes shorter until $c=0.4$ and then the period becomes longer until the stimulus is so strong that no oscillation is detectable any more. The figures in this experiment look similar to the ones from the last experiment, but with different turning points, see Fig. 4e.

We conclude from the two experiments that weak illumination (a small constant stimulus *c*) causes a shortening of the period, whereas high illumination (a larger *c*) causes a lengthening of the period. Whether and when period shortening or lengthening is observed depends on the model (fly or mammalian) and the corresponding parameters. In the fly model, the period shortening occurs only for a small range of *c* (very low illumination) while the lengthening can be seen at a wide range of “c” (low to high illumination). This is exactly what was experimentally found in flies^21^. In the mammalian model, the turning point between period shortening and lengthening happens much later, so that shortening and lengthening cover almost equal ranges of *c* (illumination). Consequently, constant light can shorten or lengthen the period of a mammal depending on the chosen illuminance. Natural light can vary between 0 and 100,000 lux. In most experiments, only a small range of illuminance was tested and consequently, either period shortening or lengthening was observed^20^. Furthermore, the responses are different in different species and most likely depend on the light-sensitivity of their eyes. Nocturnal species that usually have very light-sensitive eyes are expected to show mainly period lengthening whereas diurnal species with less sensitive eyes should predominantly show period shortening. Indeed, Jürgen Aschoff observed exactly this phenomenon and it became known as “Aschoffs rule”. Our model predicts these results. The specific mathematical argument is that models for nocturnal species have a larger $\alpha$ or $\gamma$ compared with the values used for the data plotted in Fig. 4e. Looking closer at (8) or (9), we see that due to the product of $\alpha u$ or $\gamma u$, respectively, a smaller *u* is needed in order to obtain the same coefficient in front of the term $P_{0}$ or $\exp \left( -\varrho M\right)$, respectively. This means that a weaker illuminance is needed to be right next to the turning point. The argumentation is analogous for diurnal species where $\alpha$ or $\gamma$ are smaller compared with the values used for the data plotted in Fig. 4e. We remark that the coupling constants $\alpha$ and $\gamma$ are effective parameters that contain a lot of effects that can be analyzed in more detail. For example in mammals, a closer look on the reasons of a weaker entrainment to light is that the light-receiving cells are also coupled to other cells that do not receive the light stimulus directly, as discussed in e.g.^41, (3)]^. This causes inertial effects which makes the light-receiving cells react more rigidly. The idea is similar the to experiments in section “Food entrained peripheral clocks”, where the food-entrained oscillator has to include two stimuli, one from the food intake and one from the SCN. Analogous, the light-receiving cell, which has to include light and the signals from the non light-receiving cells where of course the light-receiving also have to influence the non light-receiving cells where the inclusion is straightforward. So one approach could be to add terms in the model above that includes the effect of the other non-light receiving cells to further split the the coupling constants for the different effects and thus unfold the effects that are included in an effective coupling constant up to a purposeful level.

Furthermore, considering^21, Table III]^ and our model, we have that different organisms or mutants have different ranges of intensity where a constant stimulus causes shortening or lengthening of the period of the endogenous clock. Thus if a period increases or decreases if the organism or mutant is exposed first to constant light and then constant darkness or the other way round, depends on whether the intensity of the constant light is in the range where the corresponding graph is below the free running period or above. We stress that especially with mutants the corresponding turning point might be at a so high intensity that a external stimulus cannot be applied without causing any damage to the considered organism in order to see both behaviors of the period, that means shortening and lengthening.

We could easily adapt our model to different orders of magnitude for the illuminance by replacing *u* by $\ln \left( 1+u\right)$, for instance, adapting the corresponding parameter $\alpha$ or $\gamma$.

## Modeling central and peripheral oscillators

In this section the presented framework of external stimuli is applied to model the signaling from the SCN to the peripheral tissues to synchronize the peripheral clocks^25^. In the next experiment, we show that several oscillators can be coupled by our framework that includes external stimuli as follows. A first oscillator responds to external stimuli, for example to light as the SCN. A second oscillator receives a signal from the first oscillator that correlates to an agent involved in the first oscillator and thus this signal acts as the “external stimulus” for the second oscillator. While for the SCN (first oscillator) the external stimulus is light, the external stimulus for the peripheral clock (second oscillator) can be hormones, neuronal activity or body temperature. If one of these signals correlates with the occurrence of *per* mRNA or of the (phosphorylated) PER protein, we can couple these two oscillators for example as follows.

**Figure 5:**
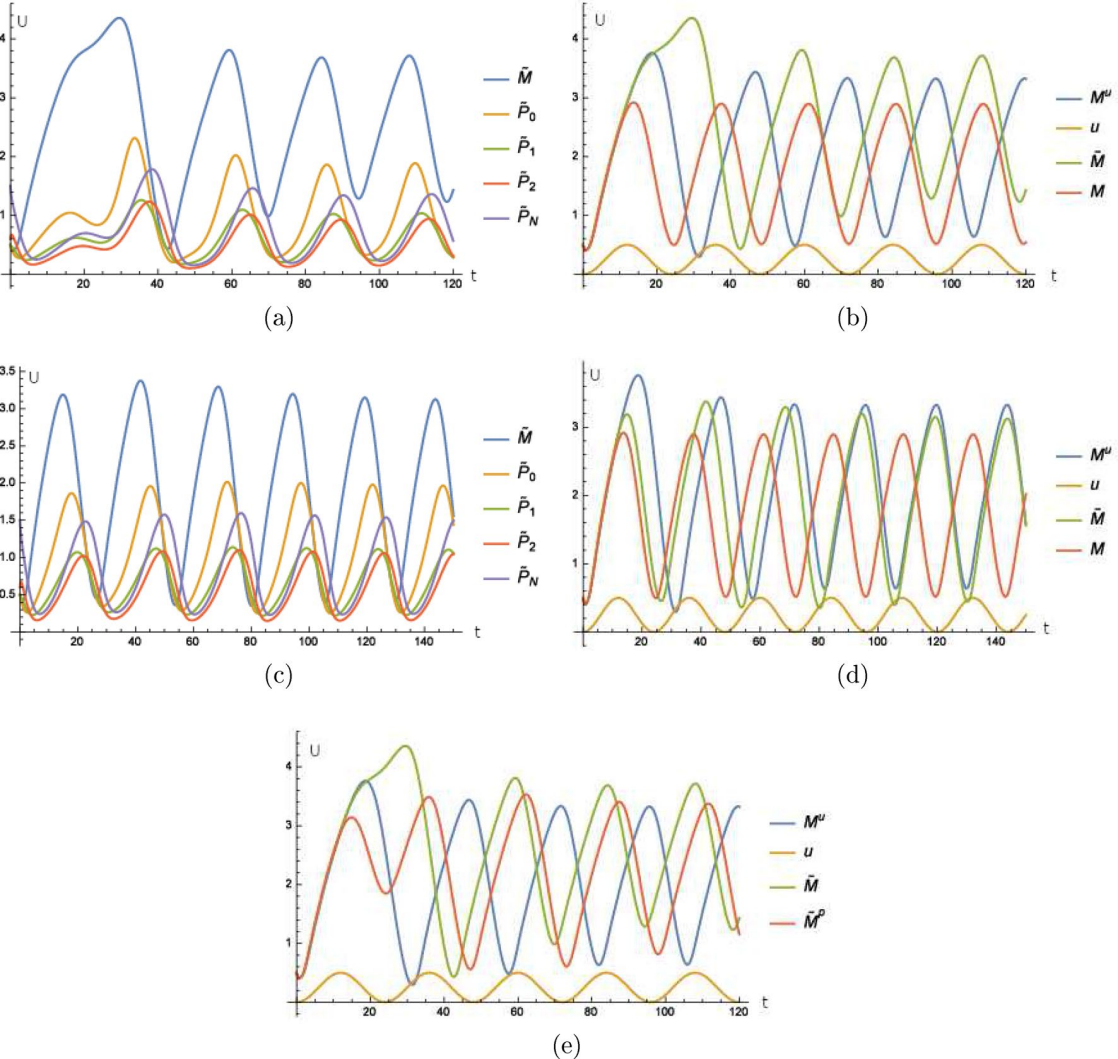
A peripheral oscillator coupled in antiphase to the molecular clock. (**a**) Time curves of the second oscillator where the agents ${\tilde{M}}$, ${\tilde{P}}_{0}$, ${\tilde{P}}_{1}$, ${\tilde{P}}_{2}$ and ${\tilde{P}}_{N}$ fulfill analog equations corresponding to (1) to (5) where (2) is replaced by (11) and ${\tilde{\alpha }}=0.5$. (**b**) Time curve of the external stimulus *u* defined in (16), of *M* calculated from the model (1) to (5), of ${\tilde{M}}$ calculated by an analogous model like (1) to (5) where (2) is replaced by (11) with ${\tilde{\alpha }}=0.5$ and of $M^{u}$ calculated from (1) to (5) where (2) is replaced by (8) with $\alpha =1$ for (16). In (**c**) and (**d**) a peripheral oscillator coupled in phase to the molecular clock. (**c**) Time curves of the second oscillator where the agents ${\tilde{M}}$, ${\tilde{P}}_{0}$, ${\tilde{P}}_{1}$, ${\tilde{P}}_{2}$ and ${\tilde{P}}_{N}$ fulfill analog equations corresponding to (1) to (5) where (2) is replaced by (12) with ${\tilde{\alpha }}=0.5$, $\beta =2$. (**d**) Time curve of the external stimulus *u* defined in (16), of *M* calculated from the model (1) to (5), of ${\tilde{M}}$ calculated by an analogous model like (1) to (5) where (2) is replaced by (12) with ${\tilde{\alpha }}=0.5$, $\beta =2$ and of $M^{u}$ calculated from (1) to (5) where (2) is replaced by (8) with $\alpha =1$ for (16). In (**e**) the time curve of the external stimulus *u* defined in (16) where $M^{u}$ and ${\tilde{M}}$ are calculated as in (**a**) and (**b**). The quantity ${\tilde{M}}^{p}$ is calculated by an analogous model as ${\tilde{M}}$ where (11) is replaced by (13) with $\alpha ^{P}=0.5$. After the transient, we have a shift of about 12.7 h between $M^{u}$ and ${\tilde{M}}$ and of about 15.8 h between $M^{u}$ and ${\tilde{M}}^{p}$. Units and dimensions: abscissa *t*: time in hours, ordinate *U*: amount of substance (*M*, $M^{u}$, ${\tilde{M}}$, ${\tilde{M}}^{P}$ mRNA, ${\tilde{P}}_{i}$, $\left\{ 0,1,2,N\right\} ,$ protein, *u* intensity of illumination).

We take the basic equations (1) to (5), where the first oscillator is sensitive to an external stimulus, that means (2) is replaced by (8). The second oscillator with its corresponding agents ${\tilde{M}}$, ${\tilde{P}}_{0}$, ${\tilde{P}}_{1}$, ${\tilde{P}}_{2}$ and ${\tilde{P}}_{N}$ is also modeled by analogous equations as (1) to (5) and is coupled to the first oscillator by also replacing (2) by

11 $$\begin{aligned} \frac{d}{dt}{\tilde{P}}_{0}=k_{s}{\tilde{M}}-V_{1}\frac{{\tilde{P}}_{0}}{K_{1}+{\tilde{P}}_{0}}+V_{2}\frac{{\tilde{P}}_{1}}{K_{2}+{\tilde{P}}_{1}}-{\tilde{\alpha }}{\tilde{P}}{}_{0}P_{0} \end{aligned}$$

with ${\tilde{\alpha }}>0$. That means there is an additional digestion of ${\tilde{P}}_{0}$ if the concentration of $P_{0}$ and ${\tilde{P}}_{0}$ is high at the same time. The concentration of $P_{0}$ is transmitted by for example some signaling pathway to the second oscillator. More specific, the further digestion of ${\tilde{P}}_{0}$ correlates to a high concentration of $P_{0}$ and ${\tilde{P}}_{0}$ at the same time. In Fig. 5a,b, we see that the second oscillator is synchronized to an antiparallel oscillation compared to the first one, that means that there is a phase shift of $\pi$ after the transient.

A parallel synchronization can be achieved by the following model

12 $$\begin{aligned} \frac{d}{dt}{\tilde{P}}_{0}=k_{s}{\tilde{M}}-V_{1}\frac{{\tilde{P}}_{0}}{K_{1}+{\tilde{P}}_{0}}+V_{2}\frac{{\tilde{P}}_{1}}{K_{2}+{\tilde{P}}_{1}}-{\tilde{\alpha }}{\tilde{P}}{}_{0}\exp \left( -\beta P_{0}\right) \end{aligned}$$

where $\beta >0$. Here, in this model, a higher concentration of $P_{0}$ causes a lesser digestion of ${\tilde{P}}_{0}$, which is transmitted by some signaling pathway for example. More specific, a lesser digestion of ${\tilde{P}}_{0}$ correlates to a higher concentration of $P_{0}$. For the discussion of replacing the term $\exp \left( -\beta P_{0}\right)$ by alternative bilinear models, see the end of “Specific organism models" below (9). Here the process can be analog where $P_{0}$ stimulates another protein which is transformed from an inactive to a stimulated species. Then the stimulated species, which is high if $P_{0}$ is low, can replace the term $\exp \left( -\beta P_{0}\right)$. For clarity we proceed with the (12) to demonstrate how to couple different small models to a big one with the idea of inputs modeled by external stimuli. In Fig. 5c,d, we see the synchronization of both oscillators with a shift of 0 after the transient.

If we take the model of two coupled oscillators mentioned above and replace (11) by

13 $$\begin{aligned} \frac{d}{dt}{\tilde{P}}_{0}^{p}=k_{s}{\tilde{M}}-V_{1}\frac{{\tilde{P}}_{0}^{p}}{K_{1}+{\tilde{P}}_{0}^{p}}+V_{2}\frac{{\tilde{P}}_{1}^{p}}{K_{2}+{\tilde{P}}_{1}^{p}}-\alpha ^{P}{\tilde{P}}_{0}^{p}P_{2}, \end{aligned}$$

where the additional decay of ${\tilde{P}}_{0}^{p}$ depends on the product of ${\tilde{P}}_{0}^{p}$ and $P_{2}$ instead of ${\tilde{P}}_{0}$ and $P_{0}$ as in (11), then we can see in Fig. 5e that the shift of $P_{2}$ compared to $P_{0}$ provides a system of two coupled oscillators where the shift of phase can have more values than just inphase courses, i.e. a phase shift of 0, see Fig. 5c or antiphase courses, i.e. a phase shift of $\pi$, see Fig. 5a. By this mechanism of coupling different oscillators, nature might create peripheral endogenous clocks which are out of phase with the clock of the SCN for a varying amount of hours. Thus they may adopt any phases that are advantageous for the physiological function of the peripheral oscillator.

## Food entrained peripheral clocks

In our next experiment, we model a similar situation as described for the liver and SCN clocks of mammals. As already described, the SCN coordinates the phases of peripheral clocks, such as that in the liver^25^. But the SCN is not the only Zeitgeber for the liver clock, food intake is another important Zeitgeber. Under normal conditions both Zeitgebers are in phase with each other. However, when food is only available outside the animal’s normal activity time, feeding can phase shift the liver clock, but not the SCN clock, so that both clocks can take an unusual phase relationship^9^. We choose the model that is used for the experiment depicted in Fig. 5c. Furthermore, we equip (12) with an additional term that models the stimulus that is caused by the food intake as follows

14 $$\begin{aligned} \frac{d}{dt}{\tilde{P}}_{0}=k_{s}{\tilde{M}}-V_{1}\frac{{\tilde{P}}_{0}}{K_{1}+{\tilde{P}}_{0}}+V_{2}\frac{{\tilde{P}}_{1}}{K_{2}+{\tilde{P}}_{1}}-{\tilde{\alpha }}{\tilde{P}}{}_{0}\exp \left( -\beta P_{0}\right) -\delta {\tilde{u}}{\tilde{P}}_{0} \end{aligned}$$

with $\delta >0$ where the external stimulus is given by

15 $$\begin{aligned} {\tilde{u}}\left( t\right) {{:}{=}}{\left\{ \begin{array}{ll} \frac{1}{4}\left( \cos \left( 2\pi \frac{t}{24}+{\tilde{\varphi }}\right) +1\right) &{} \ \hbox {if }t\le {\tilde{t}}\\ u\left( t\right) &{} \ \hbox {else} \end{array}\right. } \end{aligned}$$

with $u\left( t\right)$ defined by

16 $$\begin{aligned} u\left( t\right) {{:}{=}}\frac{1}{4}\left( \cos \left( 2\pi \frac{t}{24}+\varphi \right) +1\right) \end{aligned}$$

and ${\tilde{t}}>0$. Equation (14) induces a further digestion of ${\tilde{P}}_{0}$ by the term $-\delta {\tilde{u}}{\tilde{P}}_{0}$. More specific, the model considers the case that food intake correlates with the digestion of ${\tilde{P}}_{0}$. We choose the external stimulus *u* such that it is inphase with the SCN, that means $\varphi =0$ in (16) which is associated with day light. Now we choose ${\tilde{\varphi }}=\pi$ as food intake is supposed to happen at night instead of the day which corresponds to ${\tilde{\varphi }}=0$. That means that until ${\tilde{t}}$ food intake happens at night and after ${\tilde{t}}$ food intake is at day time.

**Figure 6:**
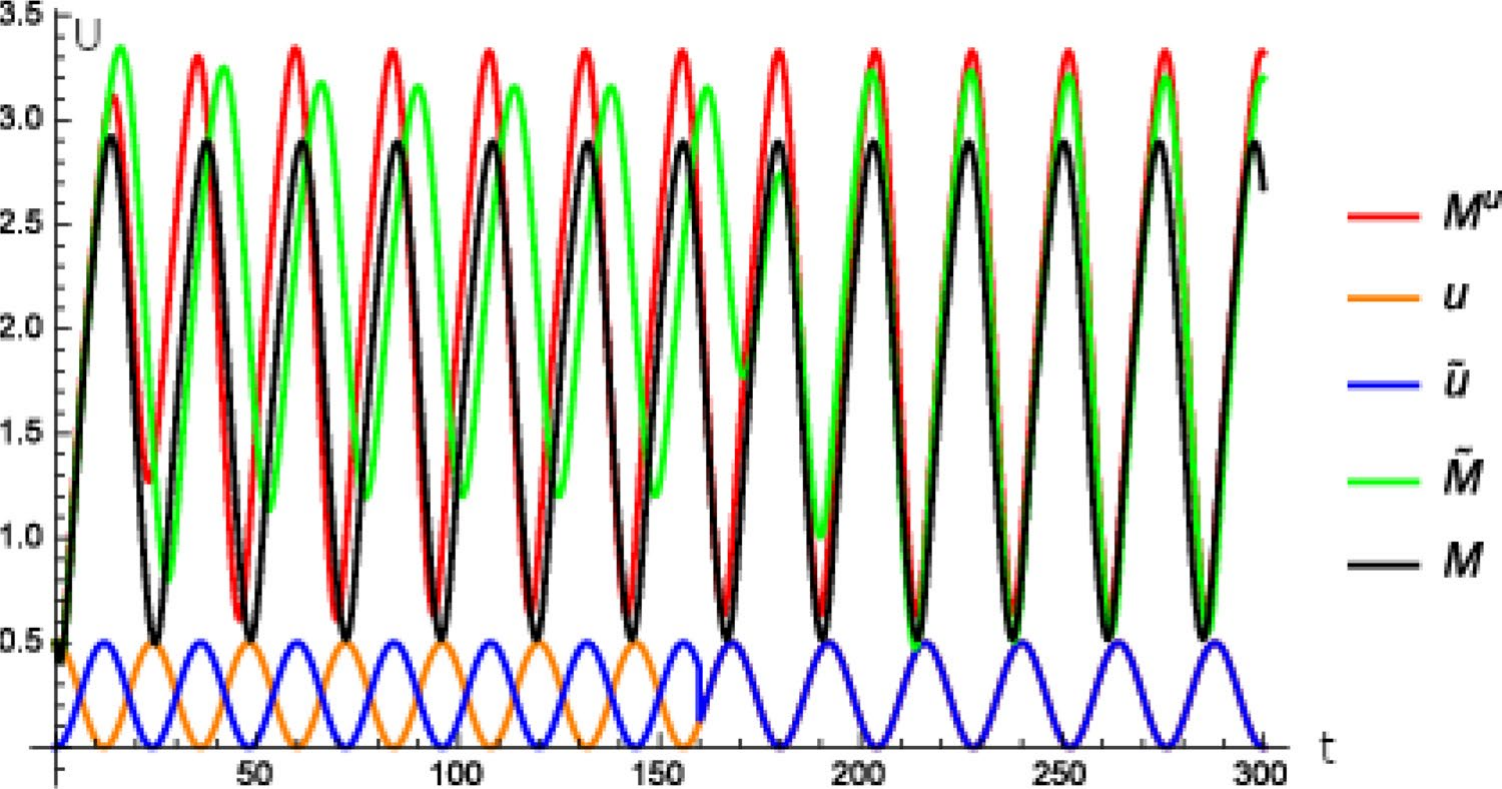
Time curve of the external stimulus *u* defined in (16) and ${\tilde{u}}$ defined in (15) with ${\tilde{t}}=160$ and ${\tilde{\varphi }}=\pi$, of *M* calculated from the model (1) to (5), of ${\tilde{M}}$ calculated by an analogous model like (1) to (5) where (2) is replaced by (14) with ${\tilde{\alpha }}=0.3$, $\beta =2$, $\delta =0.4$ and of $M^{u}$ calculated from (1) to (5) where (2) is replaced by (8) with $\alpha =1$ for (16) with $\varphi =0$. Units and dimensions: abscissa *t*: time in hours, ordinate *U*: amount of substance (*M*, $M^{u}$, ${\tilde{M}}$ mRNA, *u* intensity of illumination, ${\tilde{u}}$ intensity of signals of receptors stimulated by food intake).

In Fig. 6 we only depict the time curves for *per *mRNA to keep the figure clear for ${\tilde{t}}=160$. We see that the phase of the peripheral clock (${\tilde{M}}$) is shifted by about 6 h after the transient compared to the phase of the central clock ($M^{u}$) when food intake happens at night. When the food intake takes place again at day time as in Fig. 6 after $t=160$, i.e. there is no shift between *u* and ${\tilde{u}}$, then the peripheral clock is synchronized to the central clock again after a transient of about 1 day.

The effect on the peripheral oscillator can be interpreted as follows. All the external stimuli or signal inputs, respectively, are integrated by the peripheral clock. The optimal time point for the liver cell to be active and to fulfill the intended tasks in the body is triggered by the input and importance (strength) of the external stimuli or signal inputs received from the SCN and feeding. Reacting on different external stimuli at the same time according to a mechanism mentioned above in (14) can also be seen as making a compromise to meet all the circumstances at the same time that correspond to the simultaneously emerging external stimuli.

Notice that the closer ${\tilde{\varphi }}$ is to $\varphi$ the smaller the shift of the phases of the two oscillators is and the stronger the stimulus ${\tilde{u}}$ is the closer the shift of the phases of the two oscillators comes to the phase shift between *u* and ${\tilde{u}}$.

## Discussion

We start our discussion by briefly outlining the novelty of the present work before we give more details and relate our work to existing ones. Our simulation starts with a minimal oscillator model and it is extended by an external stimuli framework that has not been used before (see^14, 18, 35, 42, 43^). This framework allows to analyze the behavior of different external stimuli in order to demonstrate the generality and the practicability of the framework that allows easily to include external stimuli exemplified for circadian clocks and oscillator coupling. Further the novelty is especially in modeling several effects reported in experimental works just by our unified and comprehensive model. The model is mathematical consistent and uses an enzyme kinetic formalism based on the law of mass action. For this reason each equation can be investigated on its own by taking the external stimuli as an input function with given values. Then the behavior of the corresponding quantity modeled with this equation can be studied upon the external stimuli, ranging from e.g. numerical stability to effects of the external stimuli to the output values which are the values of the corresponding quantity. We do not intend to generate an animal-specific model. To achieve this, the works listed in Table 2 have to be specifically considered and the formalisms listed there added to our model, for instance the van der Pol Oscillator which is a very basal model for understanding oscillators with non-linear dynamics.

Our model provides entrainment to the phase of a periodic external stimulus and the restart of the circadian rhythm upon inhibition of transcription or translation. Different effects of external stimuli to molecular agents are investigated to set up a fly and a mammalian model that are included in our framework. The fly model has a wide range to adapt to different periods of the external stimulus while the mammalian model provides a rather short range. Behavior under constant light is investigated for both models as well as phase-response curves are calculated. It is also shown how to couple different oscillators with the presented framework to model peripheral oscillators that are coupled to a central clock and integrate and receive additional signals to process all the information incoming at once. This procedure of coupling can be extended to any model and is a helpful tool, easy to implement, to study crosstalks and network effects of coupled biological systems. In this way the modeling works module wise since sub systems can be combined together to a total system by using outputs of the sub systems as inputs for others via the proposed external stimuli framework.

The circadian clock can be modeled by a system of ordinary differential equations that stem from the theory of enzyme kinetics which shows a stable rhythmic oscillation with a period of about 24 h, see for example^14^. In our work, we aim to extend this model with additional terms modeling the effect of external stimuli like light, which causes activation or inhibition of transcription or translations as well as the degradation of proteins. We demonstrate that this model is in accordance with experimental results like entrainment of different periods in a range around 24 h, phase-response curves or changing periods under constant light. Furthermore, our model aims at the molecular effects that determine the behavior of the oscillator upon external stimuli. By external stimuli we mean any agent that effects the corresponding molecular network like light, hormones or any other chemical agent. The framework allows to investigate also various other effects reported in literature, see Table 1 for instance.

There is previous work where theoretical models are presented that describe the effects of external stimuli to circadian oscillators. In^35, 42–45^ they model the external stimuli by changing model and reaction parameters. However, we model the change of mRNA and protein levels caused by external stimuli by including additional terms into the unperturbed oscillator model. The model of^14^ exhibits a limit cycle with a period of 24 h only for a certain range of parameters. If the parameters are changed too much, the concentration of the molecular agents adjusts to a constant value as reported in^42^. Our framework does not show such a behavior because we model the external stimuli by extra terms instead of changing model parameters of the basic system that is responsible for a stable limit cycle. In our approach, we focus on effective models that decrease or increase the concentration of *per* mRNA or PER protein according to the effect of a stimulus and thus covers the effect of molecular mechanisms. By the word “effective” we mean that our model is a simple, clear and elegant one that includes only the essential information. An example can be that a time variation of an agent depends on a stimulus but the modeler can decide to what level the corresponding mechanism is unfolded in the equations to express a certain effect of an external stimulus or how different effects are summarized in a single term. Moreover, we have a straight forward way how to include experimental procedures like blocking translation as it is done in^18^, for example.

The following works^35, 42, 43, 45^ calculate phase-response curves where in^35^ also the limited entrainment to different external periods is discussed. These results are in accordance to our results, see the Supplementary material, where we focus also on the explanation of the effect that internal periods change according to permanent external stimuli. For this purpose, we use the model of^14^ with our framework of external stimuli to show that different molecular effects of the permanent stimulus result in a different behavior with respect to constant light as reported in^20^ for example (see “Constant light” under “Results”). These different effects are the degradation of PER protein and the increase of the *per* transcription. Additionally, we show (in the Supplementary material) that the endogenous clock modeled by^14^ extended by our external stimuli framework can be stopped and thus can be restarted if the corresponding stimulus declines as reported in^18^. Also by this experiment, we see that the model proposed by^14^ in combination with our framework that includes external stimuli is very robust with respect to perturbations as also for strong perturbations the oscillations with a period of about 23.7 h start again if the external stimulus declines. We also show that our model can entrain its phase to the one of an external Zeitgeber that is straightforward included in our presented framework.

It is also demonstrated in the results part how our effective model of external stimuli can be utilized to couple different oscillators, central and peripheral oscillators, see^25^ for biological examples. For the coupling mechanisms, our proposed framework of external stimuli is very useful as it focuses on the effect of the external stimuli to model not only light but also chemical agents, especially neuropeptide hormones which also have a significant effect on entrainment of peripheral clocks caused by signaling from the SCN, see for example^3, 46^. In our framework, we consider the oscillators as a macroscopic object that produces stable oscillations by molecular mechanisms whereas in^30^ the focus is on a multioscillatory approach of coupled oscillators that provides a stable output oscillation where we focus on the effects of coupled oscillators upon external stimuli.

We also assume that our system is robust against noise or in other words the noise is that small and that it does not prevent the system from producing stable oscillations. A macroscopic oscillation robust against noise can arise by coupled microscopic oscillators that can entrain each other in case of a perturbation by noise as mentioned in^12^. In^42, 47–51^ the issue is addressed with stochastic models how stable oscillations can exhibit in spite of noise. We remark that our framework can also be applied to stochastic models, in particular to the right referred. Furthermore, our framework of implementing external stimuli is not restricted to the special model presented in^14^ but can be applied to any model consisting of differential equations, like^30, 35, 36, 43^, to set up an effective model that considers the effect of external stimuli to certain agents of the network. Therefore, our framework is generic and has a high explanation power that extends the mentioned work in a new way.

Moreover, our framework also provides a principle mechanism how to explain the effects referred to points of singularity where one pulse of light can stop the oscillation and another can start the oscillation again, see^26, 40^ for further reading. Two concepts can be used for explaining this. The first possibility is to use a macroscopic model that describes a population of microscopic oscillators as a single macroscopic oscillator. To describe the singularity the corresponding equations have to contain a point of rest, sometimes called steady state, see for example^52^ for a system of differential equations having several steady states. If the external stimulus brings the system sufficiently close to that steady state, then the system converges to that point of rest as shown in^16^. This means that at this special point the values of the agents’ concentration are such that their variation with respect to time is (almost) zero and thus there are no oscillations anymore. If a second pulse of an external stimulus brings the system sufficiently away from the steady state, the oscillations start again. However, in our model based on Goldbeter’s one we have not observed such a behavior of a steady state in addition to the limit circle. A second possibility to describe the effect of singularity is to consider several microscopic oscillators whose output then is added together to a macroscopic oscillation. The key is that these microscopic oscillators are coupled inhomogeneously to the external stimulus that means that each couple constant defers a little bit. This can be for example because the cells are exposed differently to light because they are covered differently by tissue. It can be shown with our model that already five oscillators that have the same phase at the beginning get out of phase upon a 1 h pulse of external stimulus depending on its strength and at which phase time it is given. Then the total macroscopic oscillation is weaker as the microscopic oscillators have different phases caused by their inhomogeneous coupling to the external stimulus and thus the addition of all outputs provides a smaller amplitude since, for instance, one oscillator is at the lowest point of its oscillation and another is at its highest point. A second pulse brings all the microscopic oscillators to approximately the same phase again such that the macroscopic oscillation has the initial amplitude. This works because the microscopic oscillators are all influenced differently according to their phase at the time when the second pulse of the external stimulus is given such that after this pulse all the microscopic oscillators are (almost) in the same phase again. Simulations with five coupled oscillators can be found in^53, Point of singularity, page 47 ff.]^. Since the presented input-output-framework provides unified interfaces for input and output of models, it is in principle possible to construct an oscillator consisting of arbitrarily many sub oscillators with an automated script and thus simulate the points of singularity with e.g. several hundreds of single sub oscillators.

Next, we argue why single cell models or single cell data, respectively, can also be an explanation of organismic behavior: We observe evidence, see e.g. Table 1, that many organisms have an inner clock with an approximately 24 h period which influences their behavior. One can model this with any mathematical oscillator that has such a period without giving causes for the behavior, just as an observation or a description. In a next step, one may have evidence, e.g.^10, 18, 54–57^, that the expression of some genes oscillate with this period. Now one can model their interactions based on molecular mechanisms with an effective model like the Goldbeter model where the equations are motivated by kinetic equations that describe molecular reactions. Next, one observes that this model already can express also such periods and can self-containedly provide such oscillations. So now we can argue that this molecular oscillation is significant for the organismic behavior by finding such expressions in different tissue, like the brain. When investigating these issue further by looking more closely to tissues even on a cellular level, the more complex models can also be modeled with mathematical equations. In our opinion the effective model from the beginning (Goldbeter) is still useful since “effective terms” can be tissue specific unfolded step by step, going more into detail, to include all the involved agents (like genes) to a depth that seems purposefully for the considered research question. Further it can be tested whether this changes behavior or can still reproduce the observations. An effect could be e.g. longer feedback loops. However, this can lead to complex models that according to the purpose of investigation sometimes can be re-unfolded to test if the observed effect comes from the complexity of the model or rather from more abstract/effective properties of the system and thus a model with fewer agents has the same power for explaining the considered research question but is clearer. So to summarize, for the purpose of the work, we can use all oscillators that give us a 24 h period, independent of the mechanisms that motivate their mathematical structure. However, we are aware of the fact that there are use cases where the modeling of a mechanism, like gene regulation, is investigated for being able to explain the outcome of an experiment and consequently the particular structure of the corresponding equations matters. How these more complex models are easily modeled with our framework is explained above (see also Tables 1,2 and “Methods”). In particular, a realistic stochastic model for a specific organism can be even more powerful, however, this may mean to put a lot of work into details that are important for specific research questions. For general conclusions and easy adaptation to the organism of choice and regarding oscillator effects we recommend our model.

## Conclusion

We present a framework to model how external stimuli influence the circadian clock and how they can synchronize the rhythm to the external time phase. We extend the original model of Goldbeter et al.^14^ to a general framework of external stimuli. We analyze it with respect to its behavior upon different external stimuli in order to demonstrate the versatility, generality and application of our proposed external stimuli oscillator response model.

We show how to model and investigate different molecular effects of a permanent stimulus and how it results in a different behavior with respect to constant light, light–dark cycles and phase shifts by strong stimuli. In particular, we show that our external stimuli framework in combination with an effective model provides an explanation for the “Aschoff rule”, meaning the existence of intensities of light where the circadian period either lengthens or shortens.

Our effective model of external stimuli can be utilized to obtain phase response curves, couple central and peripherals oscillators and look at food entrained peripheral clocks which serves as a blueprint for module wise modeling by assemble a total biological system by sub systems using outputs of a sub system as an input for another sub system. Moreover, our framework divides a biological system into sub systems considering the process of modeling these sub systems as creating functions mapping input information to output information where the external stimuli are the interfaces. The interface consists of the input information from the environment which is given into the considered biological system and the output information of the system which evolves according to the dynamic of the biological system. The dynamic is captured in the corresponding mathematical model.

In particular, our framework provides a principle method how to investigate and explain the effects on biological oscillators referred to singularity where one pulse of light can stop the oscillation and another one can start the oscillation again by the inhomogeneous coupling of many oscillators to an external stimulus. The model allows to investigate also effects on circadian clocks such as relative coordination which means that the period of the circadian clock adapts to the period of the external stimulation in a certain range where outside this range the period of the circadian clock is not effected by the period of the external stimulation.

In summary, our model allows to elegantly achieve a semi quantitative description of diverse experimental effects on the circadian clock or a general biological oscillator upon external stimuli like light. This is comprehensively described here with a simple but effective mathematical model that contains differential equations that exhibit a solution that oscillates with a period of about 24 h and terms that increase or decrease the concentration of a corresponding molecular agent of the oscillator, depending on the intensity of the corresponding stimulus and the agent’s concentration. All scripts and the framework are available with a tutorial at https://www.biozentrum.uni-wuerzburg.de/bioinfo/computing/circadian/.

## Methods

We used the Wolfram Mathematica software, mainly the NDSolve function, to solve all the systems of ordinary differential equations of which our models consist.

### The Mathematica files

The Mathematica files from https://www.biozentrum.uni-wuerzburg.de/bioinfo/computing/circadian/ can be used for the starting point of investigations. The files are set up to solve ordinary differential equations and plots the corresponding time curves. Once the user has replaced the current model by their model, for example replace the current model equations with e.g. the ones of a model from Table 2 where the corresponding equations are referenced in the column “Model equations”, the files can immediately be used. In addition the Mathematica files can be used as a blueprint to easily build a new environment in any software that can solve ordinary differential equations numerically.

## Supplementary Information

Supplementary Information.
