# Supplementary material for: An effective model of endogenous clocks and external stimuli determining circadian rhythms
Source: Sci Rep. 2021 Aug 9;11:16165. doi: 10.1038/s41598-021-95391-y (PMC8352901; doi:10.1038/s41598-021-95391-y)
Supplement: Supplementary file 1 — Supplementary Information. [file 41598_2021_95391_MOESM1_ESM.pdf]

# Supplementary material

The Supplementary material fits to the numbering of the literature, of the pages and of the equations of the paper. The Mathematica files for the simulations can also be found in [53]. Throughout the appendix the time is plotted on the abscissa and the corresponding amount of substance is plotted on the ordinate if not otherwise stated.

# 8 Phase shifts by a strong stimulus

In this experiment, we show how our framework models the reset of the endogenous clock with a strong knock-down of the *per* mRNA ($M$) by

$$u(t) := \begin{cases} 5 & \text{if } 0 \leq t \leq t_s \\ 0 & \text{else} \end{cases} \tag{17}$$

where $t_s > 0$.

Supplementary Figure 1a and Supplementary Figure 1b show the results. We see that as soon as the external stimulus $u$ stops, then the oscillation starts again with a shift of the endogenous time's phase.

In Supplementary Figure 1c and Supplementary Figure 1d, we see that if the knock-down takes longer, the beginning of the oscillation is retarded and thus the shift of the endogenous time's phase is greater. In this way, by knocking down the *per* mRNA one can restart the endogenous time whenever needed.

Now, we show that similar results can be obtained with different models. If we model inhibition of the transcription of the *per* gene by

$$\frac{d}{dt}M = v_s \frac{K_1^n}{K_1^n + P_N^n}(1-u) - v_m \frac{M}{K_m + M} \tag{18}$$

instead of (1) while the remaining equations are given as in (2) to (5) with

$$u(t) := \begin{cases} 1 & \text{if } 0 \leq t \leq t_s \\ 0 & \text{else} \end{cases}, \tag{19}$$

$t_s = 20$, then we see a shift of the phase similar to the one in Supplementary Figure 2.

The inhibition of the translation of the *per* mRNA can be modeled by

$$\frac{d}{dt}P_0 = k_s M(1-u) - V_1 \frac{P_0}{K_1 + P_0} + V_2 \frac{P_1}{K_2 + P_1} \tag{20}$$

instead of (2) in the system (1) to (5). In Supplementary Figure 3, we see the results. The concentration of PER protein falls to zero and starts oscillating again when the translation of PER is able to start again. This is also experimentally shown in [18], specifically Figure 3 in [18], by giving cycloheximide to cultured neurons of the mammalian clock in the SCN. Cycloheximide inhibits protein synthesis at the ribosomes. As soon as the cycloheximide is washed out, the oscillations start again with all neurons having the same phase of their endogenous oscillations. Furthermore in Supplementary Figure 3, we see that the concentration of mRNA increases as there is no protein blocking the transcription of *per* mRNA.

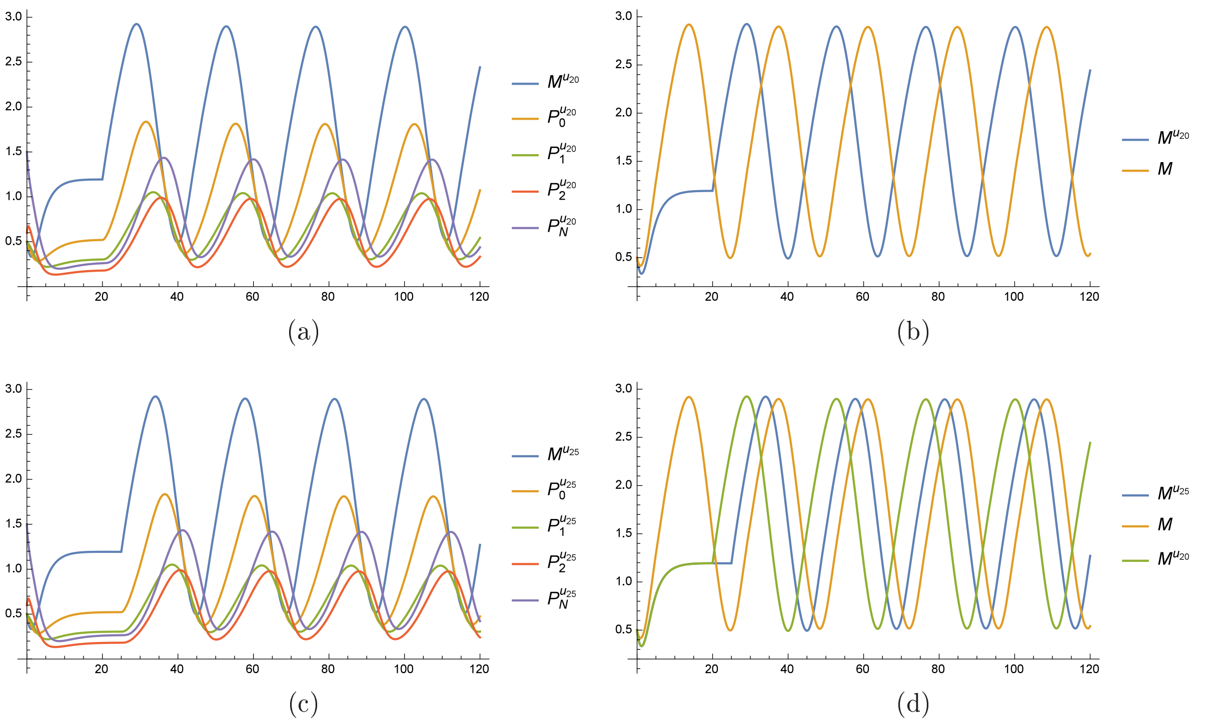


Supplementary Figure 1: The external stimulus stops the oscillations, which restart after the external stimulus has decayed. (a) Time curves of all proteins where the superscript $u_{20}$ indicates that these time curves stem from the extended model consisting of (6) and (2) to (5) for (17) with $t_s = 20$ and $\alpha = 0.05$. (b) Time curve of $M$ calculated from the model 1 to (5) and of $M^{u_{20}}$ calculated from (6) and (2) to (5) for (17) with $t_s = 20$ and $\alpha = 0.05$. Depending on the point of time when the external stimulus decays, we obtain a different shift of the molecular clock's phase in (c) and (d). The experiment is as in (a) and (b) where $t_s = 25$.

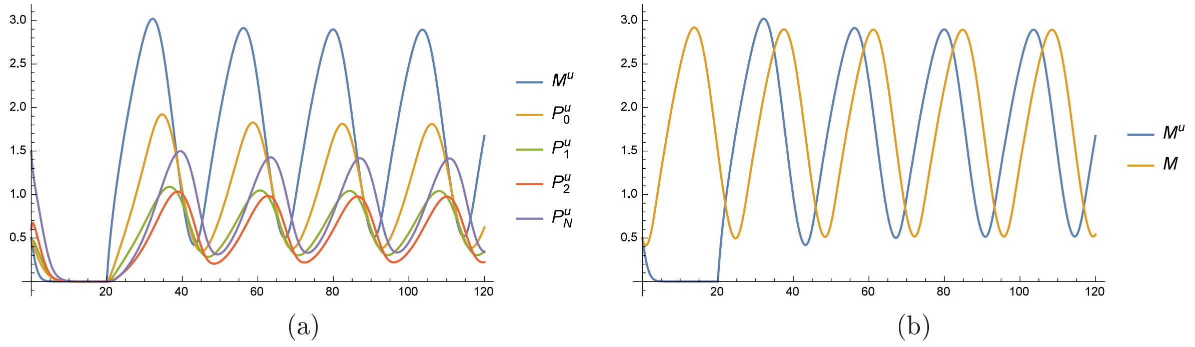


Supplementary Figure 2: After blocking the transcription of *per* gene, the molecular clock restarts as soon as the inhibition stops. (a) Time curves where the superscript $u$ indicates that these time curves stem from the extended model consisting of (18) and (2) to (5) for (19) where $t_s = 20$. (b) Time curve of $M$ calculated from the model 1 to (5) and of $M^u$ calculated from (18) and (2) to (5) for (19) where $t_s = 20$.

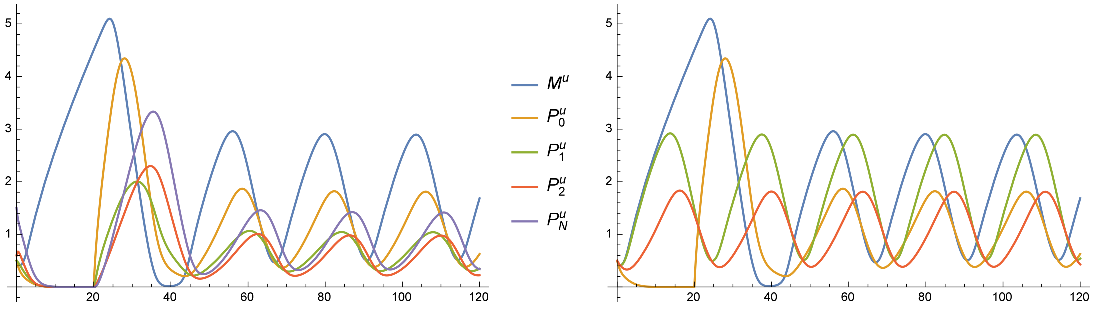


(a) (b)

Supplementary Figure 3: Blocking translation of *per* mRNA restarts the molecular clock as soon as the inhibition stops. (a) Time curves where the superscript $u$ indicates that these time curves stem from the extended model consisting of (1) to (5) where (2) is replaced by (20) for (19) with $t_s = 20$. (b) Time curve of $M$ and $P_0$ calculated from the model (1) to (5) and of $M^u$ and $P_0^u$ calculated from (1) to (5) where (2) is replaced by (20) for (19) with $t_s = 20$.

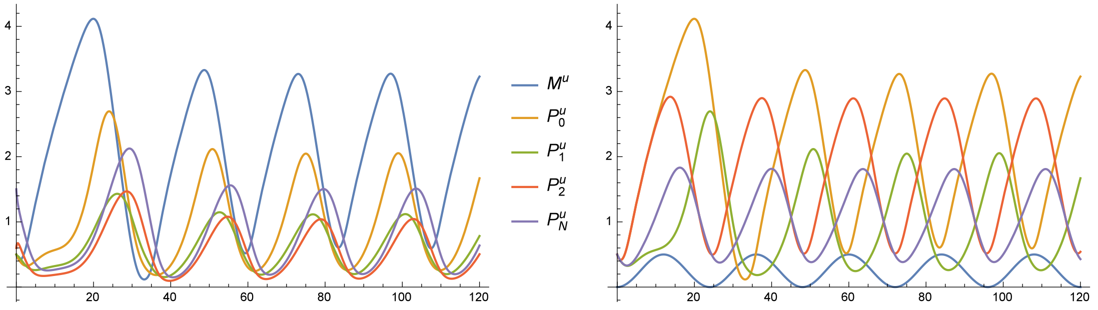


(a) (b)

Supplementary Figure 4: Inhibiting translation of *per* mRNA with a stimulus with a period of 24 hours according to (16) shifts the phase of the molecular clock. (a) Time curves where the superscript $u$ indicates that these time curves stem from the extended model consisting of (1) to (5) where (2) is replaced by (20) for (16) with $\varphi = \pi$. (b) Time curve of the external stimulus $u$ defined in (16), of $M$ and $P_0$ calculated from the model (1) to (5) and of $M^u$ and $P_0^u$ calculated from (1) to (5) where (2) is replaced by (20) for (16) with $\varphi = \pi$.

For the same model as in the last experiment but with an oscillating external stimulus given by (16) with $\varphi = \pi$, we obtain the entrainment of the endogenous clock as for the first experiment where the results are shown in Figure 2a, Figure 2c and Figure 2e. Specifically, we use the equations (6) and (2) to (5) with (7) for this experiment. See for example Supplementary Figure 4 for the synchronization of the endogenous time with the external time for a shift of 12 hours. Of course these cases could also model the degradation of the PER-TIM complex or further modifications, see Table 2.

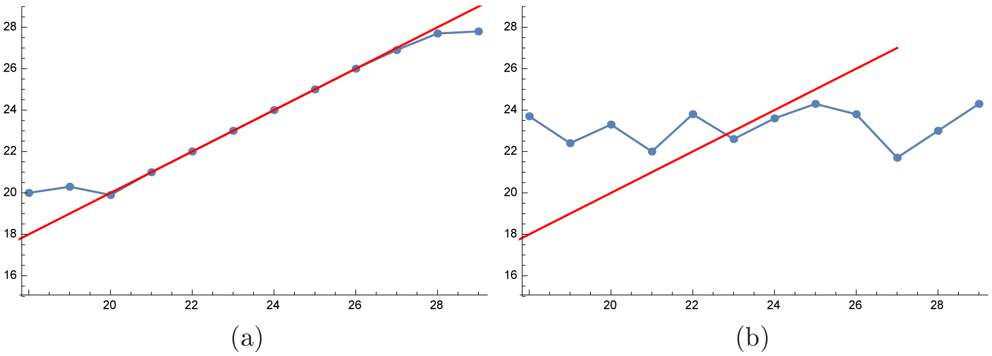


Supplementary Figure 5: On the abscissa the period $T$ of the external Zeitgeber is plotted and on the ordinate the entrained period of the endogenous clock. The red line is the ideal entrainment where the period of the external Zeitgeber is adopted. (a) Data points for the blue graph: $(18, 20.0)$, $(19, 20.3)$ , $(20, 19.9)$, $(21, 21.0)$, $(22, 22.0)$, $(23, 23.0)$, $(24, 24.0)$, $(25, 25.0)$, $(26, 26.0)$, $(27, 26.9)$, $(28, 27.7)$, $(29, 27.8)$. (b) Data points for the blue graph: $(18, 23.7)$, $(19, 22.4)$, $(20, 23.3)$, $(21, 22.0)$, $(22, 23.8)$, $(23, 22.6)$, $(24, 23.6)$, $(25, 24.3)$, $(26, 23.8)$, $(27, 21.7)$, $(28, 23.0)$, $(29, 24.3)$.

# 9 Range of entrainment

Now, we show that these systems can adapt to different periods and how this is modeled with our framework. We choose an external stimulus

$$u_T(t) := 0.06 \cdot \left(\cos\left(2\pi \frac{t}{T}\right) + 1\right) \tag{21}$$

whose period can be set to any period $T$ where $T > 0$. To exclude effects of the transient, the periods of the simulated oscillations of the *per* mRNA are determined from the peaks occurring in the range of $[170, 220]$ hours with the help of the FindArgMax function of Mathematica. We start with the model (1) to (5) where (2) is exchanged by (8) with $\alpha = 2$. We call this model the **fly model**. The range of entrainment can be seen in Supplementary Figure 5a where the red line is the ideal entrainment which means that the period of the Zeitgeber is adopted by the endogenous clock.

Now, we use the model (9) instead of (1) and still use (2) to (5) with $\gamma = 2.5$. We call this model the **mammalian model**. The parameters have different values to adapt each model to obtain a more or less realistic range of adaption to the external period. The range of entrainment is depicted in Supplementary Figure 5b where the read line also stands for the ideal entrainment.

In both cases we have a limited range of entrainment, whereby the latter is much larger in flies (8 hours), Supplementary Figure 5a, than in mammals (2 hours), Supplementary Figure 5b. This effect fits to the observed experimental data, see for example Figure 2 in [19]. We remark that the coupling constants $\alpha$ and $\gamma$ are effective parameters that contain a lot of effects that can be analyzed in more detail. For example in mammals, a closer look on the reasons of a weaker entrainment to light is that the light-receiving cells are also coupled to other cells that do not receive the light stimulus directly, as discussed in e.g. [41, (3)]. This causes inertial effects which makes the light-receiving cells react more rigidly.

In the next experiment, we show for the mammalian model that the entrained period is constant for the range of entrainment after a transient, while the periods outside the range of entrainment are only constant for a certain range followed by a range where the period changes, see Supplementary Figure 6e. These ranges follow each other and thus oscillate.This effect is known as relative

coordination, see [58] for example. The periods are determined as follows. With the Mathematica function FindArgMax, we subtract the time of the peak of $M$ in the interval $[190 + T_n, 210 + T_n]$ from the time of the peak of $M$ in the interval $[167 + T_n, 180 + T_n]$ where $T_n := 24n$, $n \geq 0$. We choose $n \in \{1, ..., 15\}$ which means that we determine the entrained period at each day. In Supplementary Figure 6, we plot the corresponding time curves of $M$ where we also see corresponding effects for the amplitude, that means constant amplitudes for the range of entrainment and oscillating amplitudes outside the range of entrainment. From these time curves the data for Supplementary Figure 6e is created.

# 10 Analyzing phase-response curves

In the presented framework phase-response curves can also be simulated. In other words our model shows that light pulses given at different times provoke different phase shifts. At certain times no phase shifts occur. We start with the fly model. If we give the light pulse when the concentration of PER protein is high, then we restart the circadian clock, see Supplementary Figure 7a and Supplementary Figure 7b, and we shift it by about eight hours. If we give the light pulse when the concentration of PER protein is low, then the clock is hardly effected, see Supplementary Figure 7c and Supplementary Figure 7d. We remark that we obtain similar figures for the mammalian model. We have a shift of the clock if the concentration of *per* mRNA is low and hardly no effect if the concentration of *per* mRNA is high. The one hour light pulse with which we perturb the endogenous clock is given by

$$u_{t_0,c}(t) := \begin{cases} c & \text{if } t \in [t_0, t_0 + 1] \\ 0 & \text{else} \end{cases} \tag{22}$$

where $c > 0$ and $t_0 \geq 0$.

In Supplementary Figure 7e, we see the phase-response curves for both models where we choose (22) with $c = 1$ and correspondingly $\alpha = 2$ and $\gamma = 2.5$. The difference of the phase is given in hours where we subtract the time of the maximum peak of the concentration of *per* mRNA from the perturbed model between $t = 85$ and $t = 95$ from the time of the maximum peak of concentration of *per* mRNA from the unperturbed model between $t = 80$ and $t = 90$. The peak of the concentration is detected by the Mathematica function FindArgMax. The phase-response curves are known from experiments, see for example [22, 23, 24]. Furthermore, the peak concentration of *per* mRNA is about 5 hours ahead of the maximum concentration of $P_0$, see Supplementary Figure 1b for example. Therefore, in the case of the mammalian model we shift $t_0$ by 5 hours such that we are in the same phase as the fly model in order to compare both phase-response curves more easily in Supplementary Figure 7e. We remark that our theoretical phase-response curves have the same qualitative course as experimental curves and even the order of magnitude of the shift fits well to experimental data.

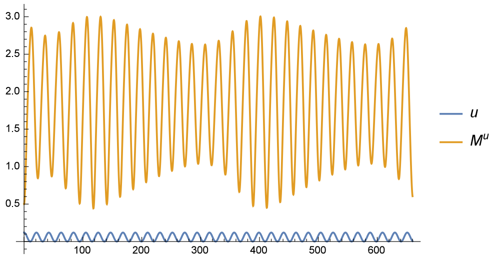


(a) $T = 21$

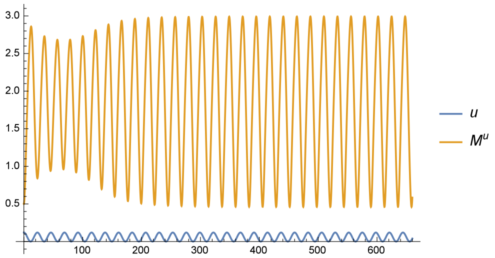


(b) $T = 23$

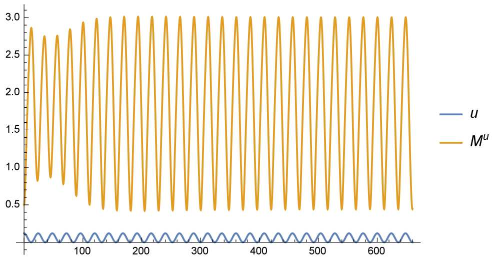


(c) $T = 24$

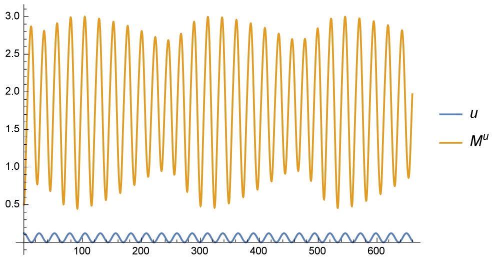


(d) $T = 26$

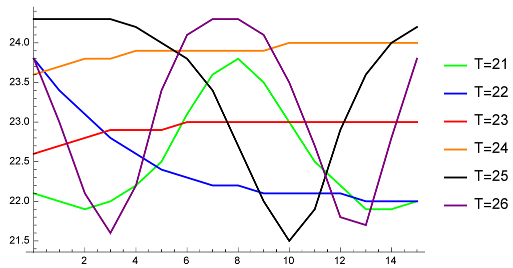


(e)

Supplementary Figure 6: In (a) to (d) we have the time curves of $M$ for the mammalian model for different periods of the external Zeitgeber. We see a constant behavior of the amplitude for the external periods that are within the range of entrainment and an oscillation of the amplitude for the periods of the external Zeitgeber that do not belong to the range of entrainment. In (e) we have the period oscillations for different periods of the external Zeitgeber for the mammalian model. The entrained periods are constant after a transient for the range of entrainment and oscillate outside the range of entrainment. On the ordinate the entrained period is plotted and on the abscissa the day $n$. The data points are as follows. For $T = 21$ $(0, 22.1)$, $(1, 22.0)$, $(2, 21.9)$, $(3, 22.0)$,$(4, 22.2)$, $(5, 22.5)$, $(6, 23.1)$, $(7, 23.6)$, $(8, 23.8)$,$(9, 23.5)$,$(10, 23.0)$,$(11, 22.5)$,$(12, 22.2)$,$(13, 21.9)$, $(14, 21.9)$, $(15, 22.0)$. For $T = 22$ $(0, 23.8)$, $(1, 23.4)$,$(2, 23.1)$, $(3, 22.8)$, $(4, 22.6)$,$(5, 22.4)$, $(6, 22.3)$, $(7, 22.2)$, $(8, 22.2)$, $(9, 22.1)$,$(10, 22.1)$, $(11, 22.1)$,$(12, 22.1)$, $(13, 22.0)$, $(14, 22.0)$, $(15, 22.0)$. For $T = 23$ $(0, 22.6)$,$(1, 22.7)$, $(2, 22.8)$, $(3, 22.9)$, $(4, 22.9)$, $(5, 22.9)$, $(6, 23.0)$, $(7, 23.0)$, $(8, 23.0)$, $(9, 23.0)$, $(10, 23.0)$, $(11, 23.0)$, $(12, 23.0)$, $(13, 23.0)$, $(14, 23.0)$, $(15, 23.0)$. For $T = 24$ $(0, 23.6)$, $(1, 23.7)$, $(2, 23.8)$, $(3, 23.8)$, $(4, 23.9)$, $(5, 23.9)$, $(6, 23.9)$, $(7, 23.9)$,$(8, 23.9)$, $(9, 23.9)$, $(10, 24.0)$, $(11, 24.0)$, $(12, 24.0)$, $(13, 24.0)$, $(14, 24.0)$,$(15, 24.0)$. For $T = 25$ $(0, 24.3)$,$(1, 24.3)$, $(2, 24.3)$, $(3, 24.3)$, $(4, 24.2)$, $(5, 24.0)$, $(6, 23.8)$, $(7, 23.4)$, $(8, 22.7)$, $(9, 22.0)$,$(10, 21.5)$, $(11, 21.9)$, $(12, 22.9)$, $(13, 23.6)$, $(14, 24.0)$, $(15, 24.2)$. For $T = 26$ $(0, 23.8)$, $(1, 23.0)$, $(2, 22.1)$, $(3, 21.6)$, $(4, 22.2)$, $(5, 23.4)$, $(6, 24.1)$, $(7, 24.3)$, $(8, 24.3)$, $(9, 24.1)$, $(10, 23.5)$, $(11, 22.7)$, $(12, 21.8)$, $(13, 21.7)$, $(14, 22.8)$, $(15, 23.8)$

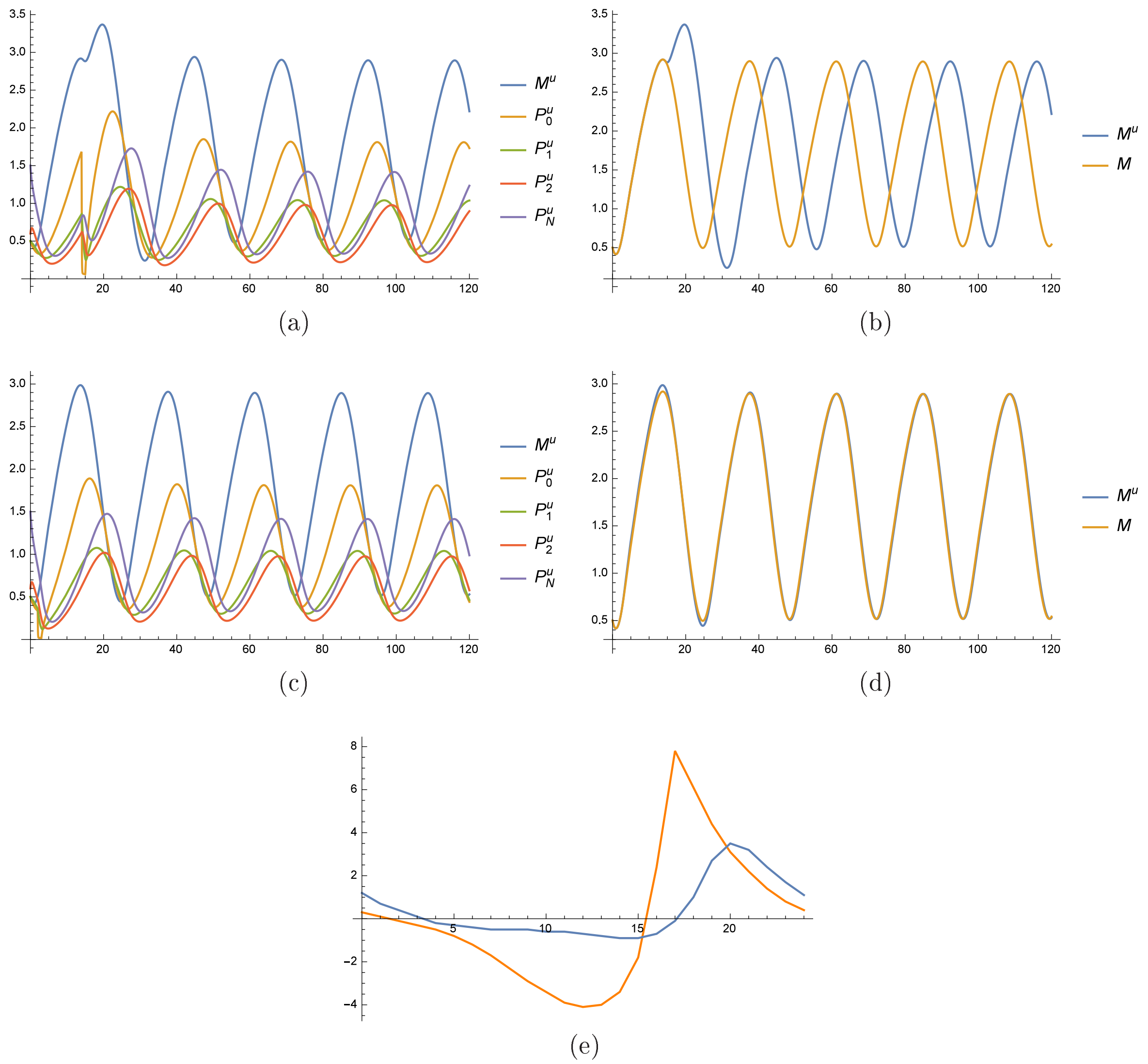


Supplementary Figure 7: A one hour light pulse causing a degradation of PER protein at a high concentration of PER protein induces a phase shift of the PER oscillation. (a) Time curves where the superscript $u$ indicates that these time curves stem from the extended model consisting of (1) to (5) where (2) is replaced by (8) with $\alpha = 1$ for (22) with $t_0 = 14$ and $c = 20$. (b) Time curve of $M$ calculated from the model 1 to (5) and of $M^u$ calculated from (1) to (5) where (2) is replaced by (8) with $\alpha = 1$ for (22)with $t_0 = 14$ and $c = 20$. In (c) and (d) a one hour light pulse does not shift the phase of the molecular clock if the pulse is given when the concentration of PER protein is low. The experiment is as in (a) and (b) with $t_0 = 2$. In (e) we plot a phase response curve. On the abscissa we have the time $t_0$ when the stimulus (22) for $c = 1$ is given where $\alpha = 2$ and $\gamma = 2.5$. The duration of the stimulus is one hour. On the ordinate we have the time difference of the maximum peak of $M$ and $M^u$, that means $\arg\max_{t\in[80,90]} M - \arg\max_{t\in[80,90]} M^u$ where $M$ is calculated from the unperturbed model (1) to (5) and $M^u$ once from the fly model, orange curve, and once from the mammalian model, blue curve.
